# Robust temporal map of human in vitro myelopoiesis using single-cell genomics

**DOI:** 10.1038/s41467-022-30557-4

**Published:** 2022-05-24

**Authors:** Clara Alsinet, Maria Nascimento Primo, Valentina Lorenzi, Erica Bello, Iva Kelava, Carla P. Jones, Roser Vilarrasa-Blasi, Carmen Sancho-Serra, Andrew J. Knights, Jong-Eun Park, Beata S. Wyspianska, Gosia Trynka, David F. Tough, Andrew Bassett, Daniel J. Gaffney, Damiana Alvarez-Errico, Roser Vento-Tormo

**Affiliations:** 1 Wellcome Sanger Institute, Wellcome Genome Campus, Hinxton, Cambridge, CB10 1SA UK; 2 Open Targets, Wellcome Genome Campus, Hinxton, Cambridge, CB10 1SA UK; 3 Graduate School of Medical Science and Engineering, Korea Advanced Institute of Science and Technology (KAIST), Daejeon, 34141 Korea; 4 Immunology Research Unit, Medicines Research Centre, GlaxoSmithKline, Stevenage, SG1 2NY UK; 5 Josep Carreras Leukaemia Research Institute (IJC), Badalona, 08916 Barcelona, Catalonia Spain

**Keywords:** Differentiation, Cell biology, Gene regulation in immune cells, Myelopoiesis

## Abstract

Myeloid cells are central to homeostasis and immunity. Characterising in vitro myelopoiesis protocols is imperative for their use in research, immunotherapies, and understanding human myelopoiesis. Here, we generate a >470K cells molecular map of human induced pluripotent stem cells (iPSC) differentiation into macrophages. Integration with in vivo single-cell atlases shows in vitro differentiation recapitulates features of yolk sac hematopoiesis, before definitive hematopoietic stem cells (HSC) emerge. The diversity of myeloid cells generated, including mast cells and monocytes, suggests that HSC-independent hematopoiesis can produce multiple myeloid lineages. We uncover poorly described myeloid progenitors and conservation between in vivo and in vitro regulatory programs. Additionally, we develop a protocol to produce iPSC-derived dendritic cells (DC) resembling cDC2. Using CRISPR/Cas9 knock-outs, we validate the effects of key transcription factors in macrophage and DC ontogeny. This roadmap of myeloid differentiation is an important resource for investigating human fetal hematopoiesis and new therapeutic opportunities.

## Abstract (web summary)

The myeloid lineage is central to homeostasis and immunity. The authors provide an atlas of human iPSC-to-myeloid cell differentiation and demonstrate that the in vitro system recapitulates yolk sac differentiation, opening new avenues to human myelopoiesis.

## Introduction

Macrophages perform a variety of functions, from tissue homoeostasis to immune surveillance and from the response to infection to the resolution of inflammation^1–4^. They originate during both development and adulthood and acquire tissue-specific functions^5^. Despite the commonalities within mammals, there are important differences between humans and rodent models^6^. Establishing and characterising the current human in vitro models is essential to fully exploit their research and therapeutic potential^7^.

During development, myeloid cells originate from at least two waves of progenitors: a first wave involving myeloid-biased progenitors from the yolk sac (yolk-sac myeloid progenitors, YSMP) and a second wave through definitive hematopoietic stem cells (HSC)^5,8^. YSMP are thought to appear during the first 2 weeks of development in humans and are responsible for producing primordial blood^5^. HSC are not generated until 3–4 post-conceptional weeks (PCW) in the gonad-aorta-mesonephros. HSC and myeloid progenitors derived from YSMP colonise the liver, making this foetal organ the main site of hematopoiesis until mid-pregnancy^9^. Later, HSC are restricted to the bone marrow, the only hematopoietic site during adulthood^10^. In mice, YSMPs generate a wide range of myeloid cells, including monocytes and neutrophils, and are thought to be the main myeloid precursors during development^11^. In humans, we have limited knowledge about the progression and regulatory mechanisms defining YSMP.

In vitro models of macrophage differentiation hold promise to not only answer these biological questions but also to become therapeutic tools, particularly immunotherapies, and for high-throughput screening, including drug testing. Macrophages derived from human induced pluripotent stem cells (iPSC) show tissue-resident phenotypes^12^ and are an attractive alternative to adult monocyte-derived macrophage cultures^13,14^. A current protocol, developed by vanWilgenburg et al., is a straightforward, feeder-free process consisting of 3 steps using constant concentrations of 1 to 3 cytokines^15^. It provides long-term, scalable production of macrophage precursors without fluorescence-activated cell sorting (FACS) since the cells of interest continuously expand and detach from the culture^16^. Despite this being an established in vitro macrophage model, the exact intermediate populations produced are unclear^12^. This restricts its applications and limits our true understanding of the cells obtained. Thus, a thorough analysis of the cell identities and dynamics emerging during in vitro differentiation is imperative to establish their likely in vivo counterparts and fully exploit this technology^17–19^.

Here, we profiled the single-cell transcriptome and open chromatin of >400k and >70k cells, respectively, during iPSC–myeloid differentiation with the vanWilgenburg protocol^15^. We uncover a wide range of cell states, their ontogeny and underlying transcription factor (TF) networks, that accurately map foetal myelopoiesis in the YS. We demonstrate the versatility of the current in vitro protocol by modifying the media used and using CRISPR/Cas9 edited iPSCs. Altogether, we demonstrate that macrophage differentiation from iPSC is a robust system to study the early stages of human myelopoiesis, and that macrophages obtained are able to acquire definitive tissue-resident identities. Our data sets can be visualised and downloaded from www.HiPImmuneatlas.org.

## Results

### iPSC-derived cells have human yolk sac myelopoiesis features

**Fig. 1:**
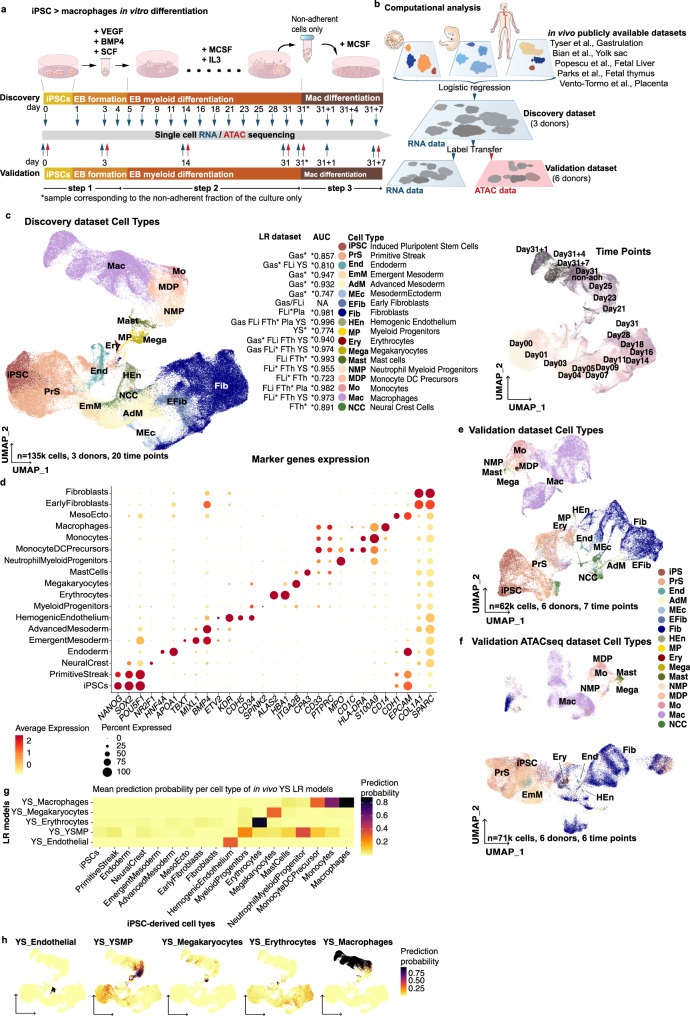
iPSC macrophage differentiation produces a range of foetal myeloid and stromal cells. **a** Schematic illustration of the in vitro differentiation protocol from iPSC to macrophages highlighting the time points sampled for scRNAseq and scATACseq profiling. Full culture well/plate was collected at each time point and at day31 the non-adherent fraction of the culture was also processed independently (day31 Non-adh). The protocol was repeated twice to generate the Discovery and Validation data sets. **b** Computational workflow diagram for cell-type annotation. Briefly, LR models were used to annotate the Discovery scRNAseq data set based on publicly available in vivo data sets of human gastrulation (Gas)^23^, yolk sac (YS)^6^, foetal liver (including skin and kidney) (FLi)^22^, foetal thymus (FTh)^21^ and placenta (Pla)^20^. Then, annotations were transferred to the scRNAseq and scATACseq Validation data sets. **c** UMAP projections of the Discovery scRNAseq data labelled by cell type. In vivo data sets supporting the cell type annotation and the area under the curve (AUC) for the best performing LR model are listed. *In vivo data set LR model of the AUC shown. (right) UMAP projections of the Discovery data set labelled by time point. **d** Dot plot showing canonical markers expression for each of the cell types. Colours depict the mean gene expression and dot size the percentage of expressing cells. **e** UMAP projections of the scRNAseq Validation data set (*n* = 62,000) labelled by cell type. **f** UMAP projections of the scATACseq Validation data set (*n* = 71,000) labelled by cell type. **g** Heatmap showing the mean logistic regression models’ predicted probabilities of the YS hematopoietic cell types^6^ for each of the cell types in the Discovery scRNAseq data set. **h** UMAP projections of the scRNAseq Discovery data coloured by the LR models’ predicted probabilities of the YS hematopoietic cell types^6^. iPSC induced pluripotent stem cell, EB embryoid body, Mac macrophage, LR logistic regression, UMAP uniform manifold approximation and projection, AUC area under the curve, ATAC assay for transposase-accessible chromatin, YS yolk sac, YSMP yolk sac myeloid-biased progenitors. Source data are provided in the Source data file.

We profiled the full differentiation of iPSC into macrophages from 6 individuals using single-cell RNA and ATAC sequencing (scRNAseq and scATACseq) (Fig. 1a and Supplementary Data 1). The differentiation protocol consists of 3 steps: (i) spin-embryoid body (EB) formation from day1 to day4, (ii) EB myeloid differentiation from day5 onwards (the latest sample used in this study is from day31), and (iii) macrophage differentiation using non-adherent cells from EB myeloid differentiation phase and lasting for 7 days, in this study we used cells from day31 (day31 to day31 + 7). To characterise the robustness of our results, we generated two independent scRNAseq data sets. The first data set (referred to as Discovery data set) included scRNAseq data from 3 donors at 20 timepoints (Fig. 1a, b). The second data set (hereafter, Validation data set) included scRNAseq and scATACseq data from 6 donors at 7 and 6 time points, respectively (Fig. 1a, b). The three donors from the Discovery data set were also used in the Validation data set, thus generating biological replicates.

After quality control, the Discovery data set contained a total of 135,000 cells (Fig. 1c and Supplementary Fig. 1). To annotate the cell types in an unbiased manner, we built logistic regression (LR) models trained on publicly available single-cell transcriptomics data sets and projected the data into our in vitro data set (Fig. 1b). We used multiple human developmental data sets to train our models^6,20–23^ (Supplementary Data 2), including a full gastrula and the main foetal hematopoietic organs: yolk sac, liver and thymus. Cell type labels were assigned based on the mean LR prediction probability of each cell cluster (Fig. 1c, Supplementary Fig. 2 and Supplementary Data 3). Marker gene expression analysis further supported the LR cell-type annotation (Fig. 1d). Cell type label transfer^24^ from the Discovery data set into the Validation and scATACseq data sets confirmed the presence of the main cell populations in all 3 data sets (Fig. 1e, f, Supplementary Fig. 1 and Supplementary Data 4).

The majority of cells at the initial EB formation stage (day1–4) matched gastrulation cell populations (Fig. 1c and Supplementary Fig. 3). We found primitive streak-like cells, emergent and advanced mesoderm, and the initial appearance of hemogenic endothelium. Despite using cytokines that induce hematopoietic mesoderm (Fig. 1a), we also observed populations related to other germ layers (i.e. neural crest and endoderm, Fig. 1c).

During EB myeloid differentiation (day5–31), the myeloid and stromal cell compartments emerged (Fig. 1c and Supplementary Fig. 3). The myeloid populations included a wide range of cell types, such as erythrocytes, megakaryocytes, mast cells, neutrophil myeloid progenitors (NMP), monocyte DC precursors (MDP), monocytes and macrophages. Being so unexpected, we validated the presence of the small population of mast cells by FACS (Supplementary Fig. 4). Most in vivo counterparts for these cell types were found in the foetal liver and thymus. However, we did not find any cluster in the Discovery data set that corresponded to the in vivo HSC found in the human developing liver (Supplementary Fig. 2B). Instead, there was a distinct cluster of myeloid progenitors (MP) expressing *CD34*, low levels of *SPINK2* and *PTPRC*, but not HOXA genes, which are required to generate definitive HSC^25,26^ (Fig. 1d and Supplementary Fig. 5A). MPs had a high prediction probability for the YSMP-trained model generated with the embryonic YS data set (Fig. 1g, h and Supplementary Fig. 2C), suggesting in vitro myelopoiesis recapitulates YS differentiation. In vivo YSMP and macrophages LR models captured more than one cell type within the in vitro data set (Fig. 1g, h). To explore this further, we performed the opposite exercise: we trained models on our in vitro cell types and projected them onto the in vivo YS data set. As expected a subset of cells within the published YSMP cluster showed a high prediction probability with the in vitro NMP-trained model and a subset of in vivo macrophages were captured by the MDP-trained model (Supplementary Fig. 5B, C). Using the LR results, we annotated the NMP and MDP cell types within the embryonic YS data set (Supplementary Fig. 5B).

To quantitatively characterise the macrophage foetal-like profile observed, we projected adult and foetal macrophages data from the human decidual–placental interface into our data set. This unique tissue setting includes both adult/maternal monocyte-derived macrophages and foetal/placental YS-derived macrophages (Hofbauer cells)^20^, thus avoiding the technical confounders of adult+foetal data set integration. The Hofbauer cells LR model had a higher mean prediction probability for iPSC-derived macrophages than any of the adult macrophage subtypes identified in the placenta (Supplementary Fig. 5D, E). As an exception, day31 + 1 macrophages presented a higher score with adult macrophages LR models (Supplementary Fig. 5D, E). This was likely because day31 + 1 macrophages showed an activated state, upregulating inflammatory cytokines such as *CXCL8*, *CCL7* or *IL1B*, (Supplementary Fig. 5F) also found on monocyte-derived macrophages in the decidua (adult/maternal tissue). Next, we used a hepatocellular carcinoma data set^27^ and projected several tumour-associated macrophages (TAMs) LR models on our iPSC-derived data set (Supplementary Fig. 5G, H). We found the foetal-like *FOLR2*+ TAMs model showed a higher prediction score among end-stage macrophages (day31 + 7) while the *SPP1*+ TAMs LR model markedly captured macrophages on the activated state (day31 + 1, Supplementary Fig. 5H). Overall, this indicated that macrophages produced in the iPSC protocol have a strong foetal phenotype, and this could be relevant for their application as in vitro TAM models.

### Trajectory analysis and underlying regulatory programmes

**Fig. 2:**
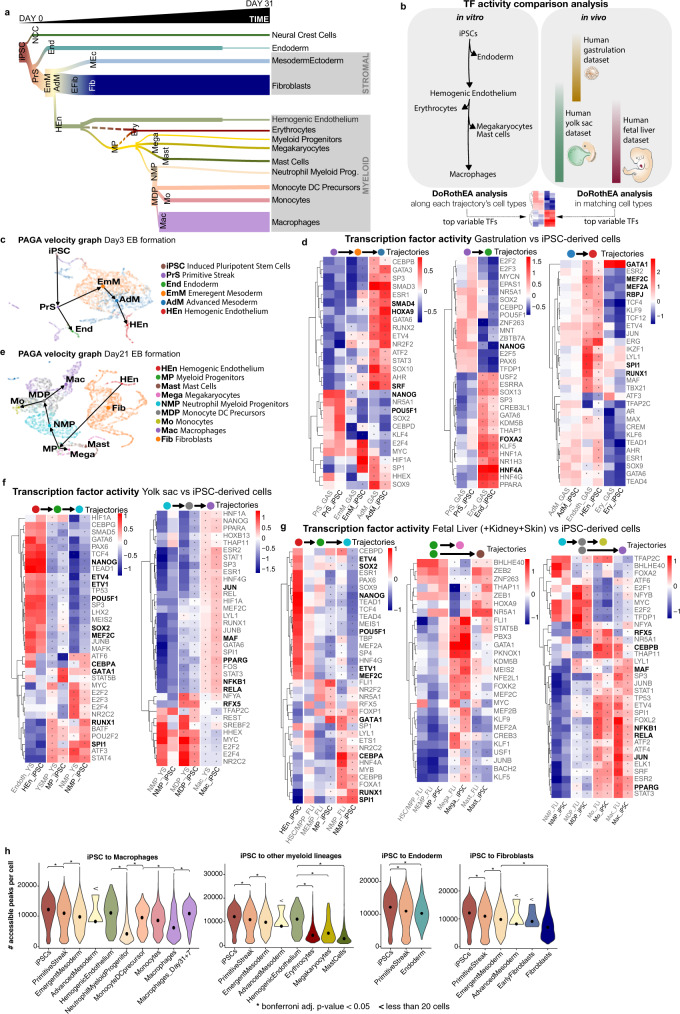
Cell population dynamics. **a** Diagram illustrating the dynamic emergence of the different cell types over the course of the in vitro differentiation protocol (Discovery data set), weaker links are shown by discontinuous lines. **b** Schematic representation of the computational workflow used to compare transcription factor (TF) dynamics in vivo and in vitro. Briefly, TF activities were computed at branching points along the in vitro differentiation trajectory (Discovery data set) and were compared to TF activities in matched cell types in the in vivo human yolk sac^6^, gastrulation^23^ and foetal liver^22^ data sets. **c** RNA velocity analysis and PAGA graph abstraction of the cells present at day3 (Embryoid body (EB) formation) of the differentiation protocol (Discovery data set) showing the developmental relationships between cell types. **d** Transcription factor activities computed with DoRothEA for the identified cell types present at day3 of the in vitro differentiation protocol and matched cell types in the in vivo gastrulation data set^23^, relevant TFs are shown in bold, asterisks highlight significantly different activity vs its progenitor, Bonferroni adjusted *p* < 0.05. **e** RNA velocity analysis and PAGA graph abstraction of the cells present at day21 (EB myeloid differentiation) of the differentiation protocol (Discovery data set) showing the developmental relationships between the cell types. **f** Transcription factor activities computed with DoRothEA for the identified cell types present at day21 of the in vitro differentiation protocol and matched cell types in the in vivo yolk sac data set^6^, relevant TF shown in bold, asterisks highlight significantly different activity vs its progenitor, Bonferroni adjusted *p* < 0.05. **g** Same analysis as **f** with matched cell types in the in vivo foetal liver, skin and kidney data set^22^. **h** Violin plots showing the number of accessible peaks per cell type in the scATACseq Validation data set. Each plot shows a distinct lineage, asterisks highlight two-tailed *t* test Bonferroni adjusted *p* < 0.05. iPSC induced pluripotent stem cells, ATAC assay for transposase-accessible chromatin. Source data are provided in the Source data file.

Myelopoiesis is shaped by transcriptional programmes, including TFs, epigenetic regulators and post-transcriptional mechanisms^28^. We set out to reconstruct the main developmental pathways underlying in vitro myelopoiesis and the transcriptional networks mediating them. The high-throughput single-cell approach used, the high density of time points collected and trajectory analysis using scVelo^29^, allowed us to reconstruct the differentiation paths giving rise to the wide range of cell types observed (Fig. 2a and Supplementary Fig. 6A). In parallel, we compared the underlying regulatory programmes mediating such transitions in vivo and in vitro. To this end, we measured TF activities by looking at the expression of consensus TF targets^30^ (Fig. 2b).

In step 1 of in vitro differentiation, iPSC differentiated into the primitive streak, which subsequently gave rise to either endoderm or emergent and advanced mesoderm (Fig. 2c). The split of primitive streak cells observed (Fig. 2c) suggested there is heterogeneity within this cell type. Later, advanced mesoderm differentiated into hemogenic endothelium, which was the precursor of myeloid cells (Fig. 2c). Primitive streak and mesoderm were transient populations that disappeared by day16, while endoderm and hemogenic endothelium were detectable until at least day31, the latest time point analysed (Fig. 2a and Supplementary Fig. 6A). The transition from mesoderm to hemogenic endothelium has also been reported during the gastrulation period^31^. TF activity analysis showed high conservation of the TF modules in these earlier stages of development (Fig. 2d and Supplementary Data 5). Pluripotency TFs activity (POU5F1, NANOG and SOX2) decreased upon differentiation into mesoderm and endoderm. As expected, in both in vivo and in vitro settings, mesoderm activated SMAD4, HOXA9 or SRF while endoderm activated FOXA2 and HNF4A. Later, hemogenic endothelium activated TFs relevant for hematopoiesis including RUNX1, SPI1, RBPJ, MEF2A and MEF2C. GATA1 was also activated in this transition but it showed the highest activation levels in erythrocytes (Fig. 2d).

In vitro, myelopoiesis started very early in the EB formation stage, but the wide range of myeloid cell types appeared almost simultaneously starting at day14, and they all endured until day31 (Fig. 2a and Supplementary Fig. 6A). The first MP appeared at day9–11 followed by erythrocytes on day11–14, and full myelopoiesis was achieved on day16–18. In addition to myeloid cells, advanced mesoderm also differentiated into an intermediate stage of early fibroblasts (day7), giving rise to fibroblasts by day9. Trajectory analysis on the sample at day21 reconstructed all myelopoiesis differentiation steps. Hemogenic endothelial cells, derived from the mesoderm, differentiated into MP, which gave rise to both megakaryocytes and NMP (Fig. 2e and Supplementary Fig. 6A). NMP gave rise to MDP, which differentiated into either monocytes or macrophages (Fig. 2e). The MDP to macrophage trajectory was consistently observed from day21 to day31 (Supplementary Fig. 6B). The differentiation pathway of macrophages through MP and bypassing the monocytes is consistent with the first waves of myelopoiesis emerging in the YS^5^.

Throughout all stages of myelopoiesis, we found high similarity between the regulatory programmes activated in vivo (YS and foetal liver) and in vitro (Fig. 2f, g and Supplementary Data 5). The transition from hemogenic endothelium to MP was characterised by the activation of multiple TFs including RUNX1, SPI1 and GATA1 (Fig. 2f, g). The MP to NMP transition showed further activation of SPI1 and CEBPA. On the contrary, a large number of endothelial TFs, such as SOX2 and the ETV family, pluripotency factors, such as POU5F1 and NANOG, or lymphoid lineage-promoting factors, such as MEF2C, were inactivated^32^. MEF2C is a TF characteristic of definitive HSC that drives lymphoid fate choice^32^. The low MEF2C activity in in vitro MPs, and in vivo YSMPs, further supported the HSC-independent features of this system (Fig. 2f).

iPSC-derived differentiation towards monocytes and macrophages also shared transcriptional programmes with its in vivo YS and foetal liver counterparts. Among the few TFs specifically activated in MDP from NMP was RFX5, which regulates MHC-II transcription and is responsible for a rare hereditary immunodeficiency^33^. We also observed activation of TFs controlling inflammatory programmes in monocytes and macrophages, such as JUN, RELA and NFKB1. In macrophages, we found key similarities related to their myeloid identity such as MAF^34^ and CEBPB^35^ and potentially underlying tissue-specific programmes, such as the alveolar macrophage programme characterised by PPARG^36,37^ (Fig. 2f, g).

Finally, we evaluated the chromatin accessibility dynamics of iPSC–macrophage differentiation using scATACseq. The number of accessible cell peaks decreased alongside the trajectories identified, suggesting a more restrictive chromatin landscape as cells differentiate. As an exception, hemogenic endothelium had a higher median of accessible peaks per cell (*n* = 11,074) than its mesoderm progenitors (‘Emergent Mesoderm’ *n* = 9759, ‘Advanced Mesoderm’ *n* = 8158). These differences were not associated with the number of expressed genes (‘Hemogenic endothelium’ *n* = 2514, ‘Emergent Mesoderm’ *n* = 3463, ‘Advanced Mesoderm’ *n* = 2725). Another exception was the macrophages after the macrophage differentiation phase (‘Macrophages_Day31plus7’), which had more accessibility peaks than the macrophages from the EB myeloid differentiation phase (‘Macrophages’ *n* = 6142 vs ‘Macrophages_Day31plus7’ *n* = 10,801) despite also having a similar number of expressed genes (‘Macrophages’ *n* = 2312 vs ‘Macrophages_Day31plus7’ *n* = 2219). Finally, NMPs had a very low number of accessible peaks (*n* = 4071), in line with the low number of genes expressed in these cells (*n* = 1782) (Fig. 2h and Supplementary Fig. 6C).

### Modifications in the last stage produce diverse macrophages

**Fig. 3:**
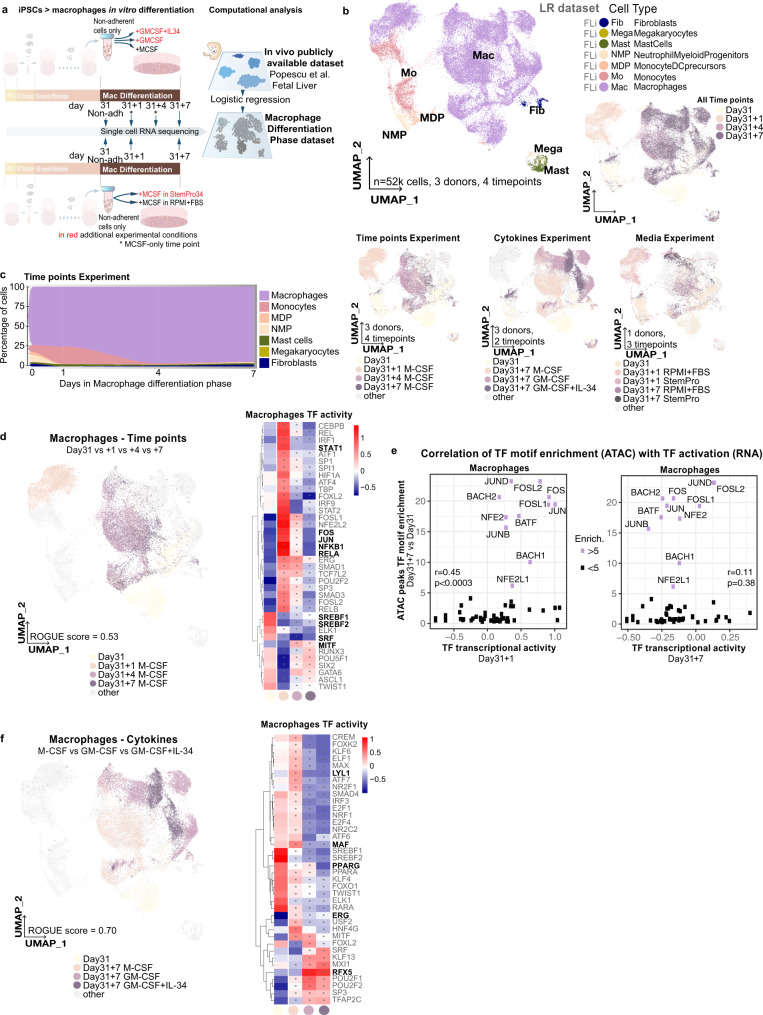
Evaluation of the macrophage phase. **a** Schematic illustration of the in vitro differentiation protocol and cell-type annotation analysis, steps before the macrophage differentiation phase are hidden. Additional experimental conditions (alternative cytokines, top, and media experiment, bottom) are highlighted in red, standard protocol conditions are in black. **b** (right) UMAP projections of the macrophage phase labelled by cell type and time points. All experiments are pooled. (right) UMAP projections highlighting the cells included in each experiment. **c** Stacked area plot of the cell type percentages in each time point. Only samples from the time points experiment (M-CSF) were included. **d** (left) UMAP projection highlighting macrophages from the time points experiment (M-CSF only) and coloured by time point. (right) Heatmap of the transcription factor activity scores calculated using DoRothEA across time points, relevant TFs are in bold, asterisks highlight significantly different activity vs day31, Bonferroni adjusted *p* < 0.05. **e** TF motif enrichment values in macrophage ATAC open peaks at day31 + 7 vs day31 plotted against TF transcriptional activity score at day31 + 1 (left) or day31 + 7 (right). Pearson correlation’s *r* and exact *p* values are shown. **f** (left) UMAP projection highlighting macrophages from the cytokines experiment and coloured by time point and cytokine cocktail used. (right) Heatmap of the TF activity scores across time points and cytokines, relevant TFs are in bold, asterisks highlight significantly different activity vs day31, Bonferroni adjusted *p* < 0.05. iPSC induced pluripotent stem cells, TF transcription factor, ATAC assay for transposase-accessible chromatin. Source data are provided in the Source data file.

Non-adherent cells at day31 were collected and plated in a fresh medium with cytokines for 7 days as step 3 of differentiation (Fig. 3a). We analysed the changes over time (time points: day31, day31 + 1, day31 + 4, day31 + 7), compared the effect of multiple cytokines (cytokines: M-CSF, GM-CSF, GM-CSF + IL-34) and tested the effect of using foetal bovine serum (FBS) vs. defined medium (media: RPMI + FBS, StemPro34). We annotated the cell types using LR from the foetal liver data set^22^ and they clustered together across experiments (Fig. 3a, b). Next, we performed a neighbourhood entropy analysis^38^ and observed that time points and media composition induced the highest diversity within macrophages (homogeneity ROGUE scores: time points 0.53, media composition 0.45 and cytokines 0.7, Fig. 3d–f and Supplementary Fig. 7A).

For the time point experiment, we analysed samples at day31, day31 + 1, day31 + 4 and day31 + 7 using M-CSF standard stimulation (Fig. 3a, b). The non-adherent cells collected at day31 from the EB myeloid differentiation phase were already mostly macrophages, alongside the main myeloid populations and a small subset of fibroblasts (Fig. 3c). By day31 + 7 there was an enrichment in macrophages, representing a total of 94.1% of cells (Fig. 3c), which was consistent with the CD14 + /CD64 + cells analysed by FACS (Supplementary Fig. 7B). We observed broad transcriptional differences between day31 and day31 + 1 macrophages, as well as between day31 + 1 and day31 + 4/7, while macrophages from day31 + 4 and day31 + 7 overlapped (Fig. 3b).

We also analysed the changes in TF activity over time^30,39^ (Supplementary Data 6). From day31 to day31 + 1, the increased activity of JUN, FOS and NFKB1 confirmed the transient but robust immune activation identified earlier (Supplementary Fig. 5F). This transcriptional activation decreased during day31 + 4 and day31 + 7 and some TF activities returned to basal levels (e.g. STAT1, NFKB1 and RELA, Fig. 3d). Despite being globally similar transcriptomically, we observed few but relevant differences between day31 and day31 + 7, including increased MITF and decreased SRF activities, which regulate phagocytosis^40,41^, in addition to decreased SREBF1 and SREBF2 activity, involved in lipid metabolism and macrophage polarisation^42,43^.

To assess whether the transient immune activation affected chromatin structure, we analysed the scATACseq macrophages data. We found that 113 TF motifs were significantly enriched on day31 + 7 ATAC peaks, compared to day31, and we obtained TF transcriptional activity scores for 55 of these (Fig. 3e and Supplementary Data 6). Importantly, the top 11 enriched motifs (enrichment score >5) at day31 + 7 corresponded to TFs that had been transcriptionally activated at day31 + 1 but were no longer activated at day31 + 7 (Fig. 3e). Indeed, the global TF motif enrichment profile at day31 + 7 correlated with TF activities at day31 + 1 (Pearson correlation: *r* = 0.45, *p* < 0.0003) but not at day31 + 7 (Pearson correlation: *r* = 0.11, *p* = 0.38) (Fig. 3e). In short, this analysis suggested that activation on day31 + 1 was transient transcriptionally but seemed to induce chromatin changes still present on day31 + 7. Therefore, earlier macrophages may represent a more naive cell state, amenable to further reprogramming in response to polarisation cues, which could have distinct applications than later macrophages.

Activated macrophages can be classified as M1 or M2 depending on whether they kickstart inflammation or resolve it, and the cytokines M-CSF and GM-CSF have been classically used to induce these phenotypes, respectively^44^. We found that specific TFs, such as MAF, ERG and LYL1, had reduced activity scores in GM-CSF vs. M-CSF macrophages (Fig. 3f and Supplementary Data 6). In contrast, RFX5 showed increased activation in GM-CSF macrophages^33^. Despite GM-CSF being largely known to promote PPARG activation^36^, this was not present in GM-CSF + IL-34 culture conditions (Fig. 3f). Of note, IL-34 is essential for the development of microglia from embryonic myeloid precursors^45^, and GM-CSF + IL34 induces the microglial phenotype on monocytes in vitro^46^. Both the knockdown and pharmacological antagonism of PPARG promotes lipopolysaccharide (LPS)‐stimulated transition from the M1 to the M2 phenotype in primary microglia, with the concomitant upregulation of markers such as CD206, TGFb and IL-4^47^.

**Fig. 4:**
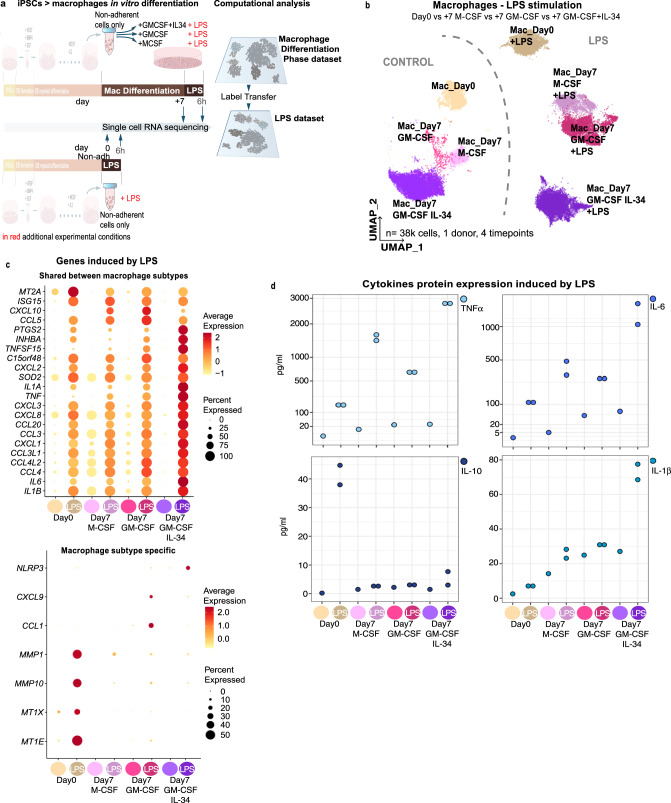
LPS stimulation of distinct subtypes of macrophages. **a** Schematic illustration of the in vitro differentiation protocol and cell-type annotation analysis, steps before the macrophage differentiation phase are hidden. Additional experimental conditions (LPS stimulation at distinct time points) are highlighted in red. **b** UMAP projections of scRNAseq analysis of LPS stimulated samples and matched controls for 4 populations of macrophages. **c** (top) Dot plot of genes overexpressed >3 log fold-change in either of the 4 LPS stimulated samples vs their control, (bottom) Dot plot of genes significantly overexpressed in 1 of the 4 populations analysed. **d** Cytokines protein expression levels in supernatants after LPS stimulation (*n* = 2) and controls (*n* = 1) for the 4 experimental conditions analysed. Media was collected from samples processed for scRNAseq (shown in **b**, **c**). Source data are provided in the Source data file.

Furthermore, we stimulated the 4 macrophage subsets with LPS for 6 h to study their TLR4 stimulation response (Fig. 4a). These included the macrophages obtained at the EB myeloid differentiation phase, as well as those obtained after the 7-day differentiation with distinct cytokines (M-CSF, GM-CSF and GM-CSF + IL-34). As a readout, we evaluated their single-cell transcriptomics profile and cytokine protein levels in the supernatant. Both analyses showed a clear distinction between control and LPS-stimulated conditions (Fig. 4b). Known LPS-induced genes (e.g. *IL6* or *TNF*) were overexpressed in all macrophage populations (Fig. 4c and Supplementary Data 7). Similarly, TNFa and IL-6 protein levels were also upregulated (Fig. 4d). Regarding LPS specific effects, macrophages from the EB myeloid differentiation phase (equivalent to day31) overexpressed metallothionein and matrix metalloproteinase genes after LPS stimulation (e.g *MT1E* and *MMP10*), along with increased IL-10 protein levels (Fig. 4c, d). Macrophages after 7 days in GM-CSF showed transcriptomic upregulation of *CCL1* and *CXCL9* cytokines with LPS (Fig. 4c). Macrophages after 7 days in GM-CSF + IL-34 upregulated the inflammasome complex member *NLRP3*, which is involved in the cleavage and activation of IL-1B. Accordingly, *IL1B* was upregulated both at the RNA and protein levels in this macrophage subset upon LPS stimulation (Fig. 4c, d).

Finally, we tested whether using a defined medium (StemPro34 serum-free media, SP-SFM) at this step would prevent the transient macrophage activation. TF activity analysis showed SP-SFM induced similar activation signals (e.g JUN, FOS and NFKB1). However, most TF activities were only partially recovered by day+7, including PPARG activity which did not significantly decrease from day+1 level. One additional difference was the maintained activity of the SREBF1 and SREBF2 TFs in SP-SFM, which link lipid metabolism to macrophages' inflammatory response (Supplementary Fig. 7A and Supplementary Data 6). These findings showed how media affects macrophage metabolism and function, which can expand the use of this model^42,43^.

### GM-CSF + FLT3L iPSC differentiation produces dendritic cells

**Fig. 5:**
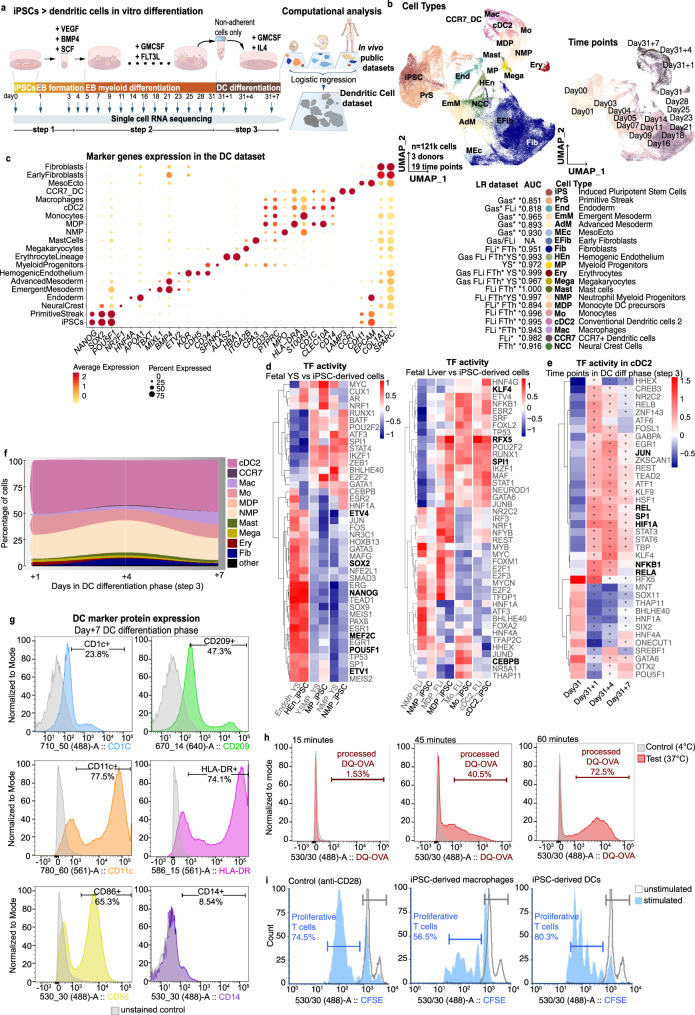
Modification of differentiation cytokines produces dendritic cells. **a** (left) Schematic illustration of the in vitro differentiation protocol from iPSC to dendritic cells highlighting the time points when samples were collected for scRNAseq profiling. (right) Computational workflow diagram for cell-type annotation. **b** UMAP projections of the scRNAseq data labelled by cell type (left) and time point (right). In vivo data sets supporting the cell type annotation and the area under the curve (AUC) for the best performing LR model are listed. *In vivo data set LR model of the AUC shown. **c** Dot plot of canonical marker genes expression for each cell type. **d** Transcription factor (TF) activities computed with DoRothEA for the identified cell types present at day21 of the in vitro differentiation protocol and matched cell types in the in vivo yolk sac data set^6^ and foetal liver, skin and kidney data set^22^. Relevant TFs are in bold. **e** TF activities computed with DoRothEA for cDC2 identified at the last 4 time points of the differentiation protocol (step 3). Relevant TFs are in bold, asterisks highlight significantly different activity vs day31, Bonferroni adjusted *p* < 0.05. **f** Stacked area plot showing the proportions of the major cell types from day31 + 1 to day31 + 7. **g** Flow cytometry histograms showing the protein levels of cDC2 marker genes and CD14 as a negative marker in non-adherent cells at the end of the DC differentiation phase (day31 + 7), matched unstained controls are shown in grey. **h** Flow cytometry histograms for BODIPY™ FL DQ-ovalbumin processing by non-adherent cells at the end of the DC differentiation phase (day31 + 7) incubated for 15, 45 and 60 min at 37 °C. In grey are matched samples kept at 4 °C as a negative control. **i** Flow cytometry histograms for a T cell activation assay, peaks with lower CFSE signal than unstimulated T cell negative control (in grey) correspond to proliferative/activated T cells by the presence of anti-CD28, iPSC-derived macrophages or iPSC-derived DCs. Plots shown are representative of two donors and two independent experiments. Source data are provided in the Source data file.

Conventional dendritic cells (cDC) present antigens to T cells and act as messengers between innate and adaptive immunity^48^. Protocols to induce DC differentiation in vitro are based on supplementing factors, including GM-CSF and FLT3L, that act cooperatively on cell precursors to drive cDC generation^49,50^. To produce iPSC-derived DCs we used GM-CSF + FLT3L in the EB myeloid differentiation phase (instead of M-CSF + IL3) and GM-CSF + IL4 in the last phase of differentiation on non-adherent cells from day31 (instead of M-CSF) (Fig. 5a). We then performed single-cell transcriptomic analysis and annotated cells using LR classifiers that were trained on gastrulation^23^, YS^6^ as well as foetal liver and thymus^21,22^ data sets (Fig. 5a, Supplementary Fig. 8 and Supplementary Data 8).

GM-CSF + FLT3L followed by GM-CSF + IL4 stimulation produced DCs with a transcriptomic profile similar to cDC2 and a small DC population resembling CCR7 + DCs. These populations were exclusive to this protocol and were not found in the Discovery data set (M-CSF + IL-3 followed by M-CSF, Fig. 1c). All other cell types in the myeloid and stromal cell compartments, and their temporal dynamics (Supplementary Fig. 9A), remained the same as the macrophage protocol (Figs. 5b and 1c). Marker gene expression analysis supported the cell type annotations, and cDC2 expressed bonafide DC markers (e.g., *HLA-DR*, *CD1C*, *CLEC10A*)^48^ (Fig. 5c). The identity of cDC2 was further explored using LR models built on adult tonsil cDC2s and two subsets of monocyte-derived DCs (moDCs) from adult ascites^51^. To match the cell types present in the adult data set we created a mixed manifold including the samples from day31 to day31 + 7 from both macrophages and DC protocols. The tonsil cDC2s LR model showed the highest prediction probability on the cells identified as cDC2s on the mixed manifold (tonsil cDC2 model AUC 0.837, Supplementary Fig. 9B, C), thus supporting our annotation. One of the moDCs subsets from ascites, which showed blood cDC2 features, overlapped with our in vitro cDC2 cells with a lower AUC (blood cDC2-moDC AUC 0.756 Supplementary Fig. 9B, C). This result demonstrated our in vitro cells have cDC2 features, although we cannot fully discard a moDC identity. The small subpopulation annotated as CCR7 + DCs showed a strong and specific prediction using a foetal thymus^21^ CCR7 DC LR model (Supplementary Fig. 8) and the expected marker expression (e.g., *CCR7*, *LAMP3* and lack of *CD14*; Fig. 5b, c and Supplementary Fig. 9D). This result showed the ability of iPS-derived DCs to adopt known activated DC profiles described in vivo^52^. Finally, cells obtained with the DC protocol showed dendrite-like structures in contrast to iPSC-derived macrophages (Supplementary Fig. 9E).

As in the presence of M-CSF + IL-3 (macrophage protocol) (Fig. 2f, g), myeloid cells appearing in the presence of GM-CSF + FLT3L (DC protocol) activated similar TFs when compared to their YS and foetal liver in vivo counterparts (Fig. 5d and Supplementary Data 9). Moreover, in vitro cDC2s activated PU.1 (SPI1 gene)^53^ and KLF4^54^ TF networks relevant for in vivo cDC2 identity (Fig. 5d). We also observed increased RFX5 activity, which regulates MHC II gene expression^33^. A recent study postulated a role for CEBPB in the control of DC maturation and later stages of DC commitment^55^. Our results showed reduced CEBPB activity in cDC2 cells compared to monocytes (in vivo and in vitro, Fig. 5d), which indicates that the in vitro phenotype shares features with a functionally mature DC subset characterised by upregulation of costimulatory and MHC class II molecules.

We analysed step 3 of the DC protocol in detail, from the non-adherent cells produced during EB myeloid differentiation until the end of DC differentiation phase. A mean of 47.5% (standard deviation = 3.67) of the cells produced in the last three time points were cDC2 cells (Fig. 5f). Thus, this differentiation was less efficient than the macrophage protocol, where macrophages represented 94.1% of cells by day31 + 7 (Fig. 3c). In contrast to what was observed with macrophages, the proportion of cDC2 remained stable (Figs. 3c and 5f). Regarding the TF activity analysis, the in vitro activation of cDC2 and macrophages induced shared regulatory programmes, including activation of NFKB1, RELA or JUN on day+1 of this phase (Figs. 3c and 5e and Supplementary Data 9). Interestingly, the particular profile of TF networks induced in cDC2 on that day (including JUN, REL, SP1 and HIF1A) did not clearly return to basal levels by day31 + 7 (Fig. 5f and Supplementary Data 9). This was in contrast to what was observed in the macrophage differentiation phase (Fig. 3d and Supplementary Data 6).

To confirm the DC phenotype of the iPSC-derived cells in our new protocol, we analysed canonical DC markers by FACS and functionally interrogated the cells using an antigen processing (DQ-OVA) and T cell activation assays. We observed positive cells for DC markers (CD1C, CD209, CD11c, HLA-DR, CD86) and low levels of the macrophage marker CD14, thus validating the DC phenotype^56^ (Fig. 5g and Supplementary Fig. 9F). Functionally, DQ-OVA antigen processing is higher in adult moDCs at earlier time points (15 to 30 min), whereas cDC processing capacity peaks at 60 min^57^. Accordingly, iPSC-derived cDC2 showed DQ-OVA processing at longer time points only (45 and 60 min) (Fig. 5h). While this time distinction is not commonly used to distinguish moDCs from cDC2s, it shows that the antigen processing behaviour of our iPSC-derived DCs resembles that of cDC2s. Finally, the cells produced at the end of the DC and macrophage differentiation were co-cultured with human CD4 + T cells purified from peripheral blood mononuclear cells. iPSC-derived DCs showed a stronger ability to induce T cell proliferation (Fig. 5i). These results demonstrated the DCs generated in our newly developed protocol were transcriptionally and functionally similar to in vivo cDC2, and could be used as a model to study DC function in vitro.

### GWAS immune phenotype genes shape iPSC-derived myeloid cells

Dysfunctional myeloid differentiation and signalling can lead to immune-related disorders^58^. To dissect potential myeloid contributions involved in these pathologies, we selected four genes linked to immune-related GWAS hits (*ICAM1*, *LSP1*, *PRKCB* and *ZEB2*) based on the existing literature. The *ICAM1* and *LSP1* loci contain SNPs linked to autoimmune inflammatory diseases by GWAS (https://www.ebi.ac.uk/gwas/home) and interact with each other^59^. *PRKCB* is a protein kinase associated with inflammatory diseases and blood cell counts in GWAS and is involved in myeloid DC differentiation^60^. Finally, *ZEB2* is found in GWAS for blood phenotypes, and regulates hematopoiesis in mice^61^ and DC cell fate decisions^62^. To study their involvement in myeloid differentiation and identity, we generated knock-out (KO) iPSC lines using CRISPR/Cas9 in one of our donor cell lines (kolf_2) (Supplementary Fig. 10).

**Fig. 6:**
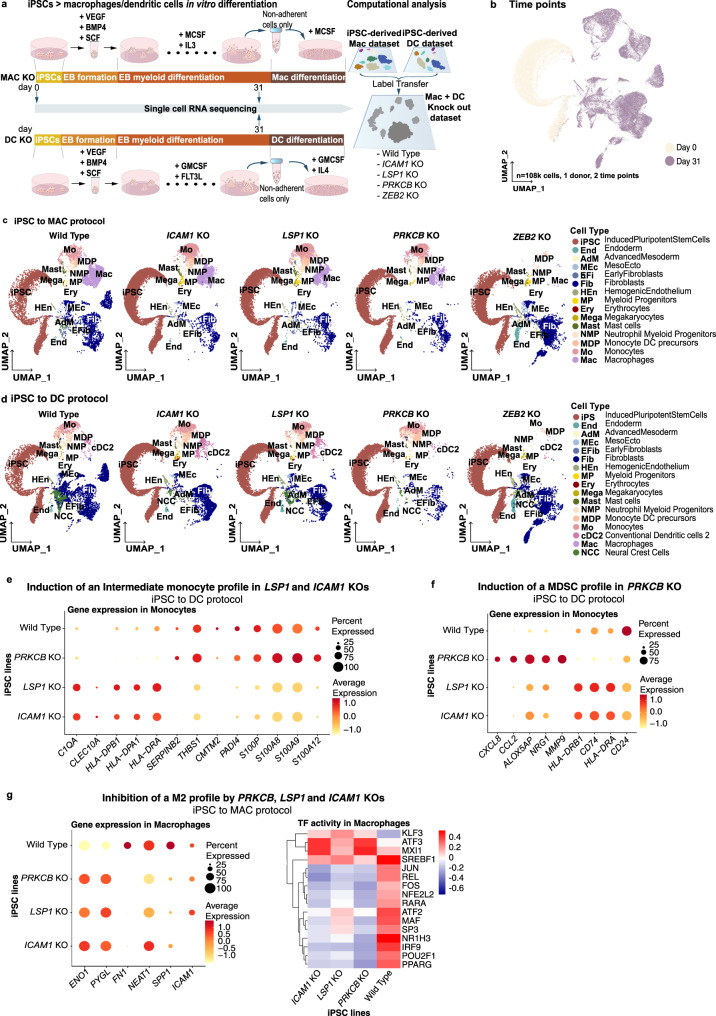
Effect on macrophage differentiation of *ICAM1*, *LSP1*, *PRKCB* and *ZEB2* KO. **a** (left) Schematic illustration of the in vitro differentiation protocols from iPSC to macrophages (MAC, top) or dendritic cells (DC, bottom) used to evaluate the effects of *ICAM1*, *LSP1*, *PRKCB* or *ZEB2* knock-outs (KO). Samples were collected at day0 and day31 of the protocols and profiled with scRNAseq. (right) Computational workflow diagram for cell type annotation. Briefly, cell type annotations were transferred from scRNAseq data of the macrophages (Discovery data set) and DC protocols described in the previous sections. **b** UMAP projections of scRNAseq data from both KO protocols labelled by time point. **c** UMAP projections of scRNAseq data generated from the iPSC-to-macrophages KO protocol (one UMAP per KO and wild type) coloured by cell type. **d** UMAP projections of scRNAseq data generated from the iPSC-to-DC KO protocol (one UMAP per KO plus wild type) coloured by cell type. **e** Dot plot showing the average expression of intermediate monocyte–associated genes in the monocytes produced by each KO and the wild type in the iPSC-to-DC protocol. **f** Dot plot showing the average expression of genes associated to myeloid-derived suppressor cells in the monocytes produced by each KO and the wild type in the iPSC-to-DC protocol. **g** (left) Dot plot showing the average expression of M2-associated genes in the macrophages produced by each KO and the wild type in the iPSC-to-macrophages protocol. (right) Transcription factor activities computed with DoRothEA for macrophages produced by each KO and the wild type.

KO iPSC lines were differentiated into macrophages and DC alongside wild-type (WT) isogenic lines. Single-cell transcriptomic analyses were performed at day0 (iPSC stage) and day31 (EB myeloid differentiation phase) (Fig. 6a, b). Though we observed all cell populations in all conditions (Fig. 6c, d), some of the KOs affected the cell type proportions (Supplementary Fig. 11A). As expected, knocking out *ZEB2* reduced myeloid cells to 5.5% versus 65.6% in WT lines in the macrophage protocol (12-fold decrease), and 2.1% versus 20.4% in WT lines in the DC protocol (10-fold decrease) (Supplementary Data 10). The overall reduction in cell numbers in the *ZEB2* KO vs the other lines suggested that *ZEB2* absence either blocked myeloid differentiation or induced apoptosis as described for myeloid leukaemic cells^63^. On the contrary, *PRKCB* KO increased the proportion of myeloid cells in the DC differentiation protocol (20.4% in WT versus 78% in *PRKCB* KO, fourfold increase) (Supplementary Data 10). The other KOs did not markedly influence myeloid cell proportions.

Some myeloid cell types showed transcriptomic differences between KO lines. *LSP1* and *ICAM1* KOs monocytes from the DC protocol had a transcriptomic profile with intermediate monocytes features. They were characterised by the upregulation of the HLA genes and downregulation of the S100 gene family^64^ (Fig. 6e). Interestingly, KO monocytes from the macrophage protocol did not show this profile (Supplementary Fig. 11B). An increased population of intermediate monocytes has been described in many autoimmune diseases such as active Crohn’s disease^65^ and rheumatoid arthritis^66^. Monocytes from the *PRKCB* KO cell line after the DC differentiation protocol had a myeloid-derived suppressor profile, including low expression of *HLA-DR* and *CD74* with high levels of *CCL2* and *MMP9*^67–70^ (Fig. 6f). This was consistent with the observation that myeloid-derived suppressor cells have decreased levels of *PRKCB*, which dampens DC differentiation and function in vivo^60^.

Macrophages generated from iPSCs deficient in *PRKCB*, *LSP1* or *ICAM1* exhibited a mixed anti-inflammatory and anti-fibrotic phenotype. KO macrophages upregulated the suppressors of the NFkB-dependent inflammatory pathway KLF3^71^ and ATF3^72^ (Fig. 6g). They also decreased the expression of M2 profibrotic phenotype genes (e.g. *FN1*, *GRN* and *SPP1*, Supplementary Data 11), as well as decreased activity of M2-promoting transcription factors (e.g. MAF^34^ and PPARG^73^, Fig. 6g). We observed a connection between *PRKCB*, *LSP1* and *ICAM1*, as *ICAM1* was downregulated in *PRKCB* and *LSP1* KOs (Supplementary Data 11). This suggested that the 3 genes are part of a regulatory network that fine-tunes macrophage phenotype and represses tissue healing both by promoting REL/p65-mediated inflammation and controlling the expression of profibrotic M2 genes. Notably, these KO lines generated a macrophage population with regenerative potential due to the concomitant suppression of the tissue remodelling programmes (Fig. 6g) and the NFkB pathway. Our data illustrate how such broad defects in macrophage polarisation could impair the proper resolution of inflammation, leading to in vivo autoimmune inflammatory disorders^74^.

Altogether, we found deletion of genes associated with autoimmunity mediated early hematopoiesis (e.g. *ZEB2*) and influenced the inflammatory potential (e.g. *ICAM1*, *LSP1* and *PRKCB*) of iPSC-derived myeloid cells, coinciding with the expected phenotype. Therefore, iPSC-differentiation protocols can be powerful tools to functionally interrogate GWAS hits and other genes of interest in vitro.

## Discussion

The full characterisation and assessment of the robustness, accuracy and efficiency of in vitro protocols are essential to utilising them as models for disease as well as leveraging them in the search for novel therapeutic targets. Myeloid cells are central to immunity and participate in major inflammatory and autoimmune disorders. Experimental protocols to generate macrophages that are easy to replicate and amenable to scaling up are paramount to studying human macrophage ontogeny, genetics and function in health and disease. Here, we profiled more than 470k single cells across a commonly used, straightforward differentiation process from human iPSC to terminally differentiated macrophages. We reconstructed, using cell trajectories, the in vitro sequence of events, which parallel foetal hematopoiesis prior to the establishment of HSC. Moreover, we show this protocol is a valuable resource in studying different macrophage cell states, multiple myeloid populations, including erythrocytes, megakaryocytes and mast cells that spontaneously arise, as well as DC, induced by adjusting the media composition. Finally, we used this model to interrogate the functional effect of genes associated with inflammatory and autoimmune disorders and interpreted the results in relation to their in vivo counterparts.

To quantitatively assess the accuracy of our in vitro models, we used machine learning tools. We built LR models trained on scRNAseq data from developmental atlases mapping the formation of the immune system and projected them onto the in vitro data sets. The computational framework we have established could be adapted to annotate multiple iPSC differentiation protocols. Following this strategy, we found that the initial phases of iPSC–macrophage differentiation faithfully recapitulate YS foetal hematopoiesis and generate foetal-like *FOLR2*+ macrophages. The lack of an HSC cluster in our data, the activation of master regulator RUNX1 in the endothelial-to-hematopoietic transition^75^, and the lack of expression of HOXA genes in the myeloid progenitors^25^ all suggest the iPSC-derived macrophages protocol closely recapitulate YS differentiation prior to the establishment of definitive hematopoiesis. Thus, we propose this model as a unique system for interrogating the early stages of hematopoietic differentiation in humans, which are largely unexplored.

Our study shows that iPSC–macrophage differentiation generates a wide range of myeloid cells and presents a detailed list of TFs that mediate the generation of distinct myeloid progenitors in vitro. We also observed that EB stimulation with GM-CSF and FLT3L produces cell populations resembling cDC2^48^. However, these in vitro DCs may also be related to moDCs, due to the presence of monocytes in the culture and the resemblance with an in vivo moDC subset, which shows features of blood cDC2s^51^. Additionally, a small subpopulation of DCs in the culture recapitulates the CCR7+ in vivo activation state^52^, highlighting the functional potential of iPSC-derived DCs. Nevertheless, the protocol for DC generation had a lower efficiency than the macrophages protocol, which is consistent with the known bias of YSMP towards macrophages^6,20–23^.

Interestingly, we found erythrocytes in our data following a decline in RUNX1 and SPI1 TF activity^76^ and a rise in GATA1 activation^77^. The in vitro iPSC differentiation of erythroid-lineage cells has relevant biomedical implications in the study and treatment of anaemias^78^. Monocytes are also generated during differentiation but, despite the complexity and limitations of trajectory analyses^79^, our analyses consistently indicate that produced macrophages bypass the monocyte stage. It is tempting to speculate that monocytes from this protocol can also be differentiated into macrophages and polarised to specific functions^80,81^. However, the lack of surface proteins uniquely expressed in monocytes (vs macrophages) makes this study more challenging. Future work should evaluate if the origin of macrophages imprints on their function.

Macrophages are a heterogeneous cell type and epigenetic states are instrumental in the generation of functional and phenotypic diversity^81–83^. Nevertheless, proper models to study this heterogeneity are missing. Here, we demonstrated that exposure to M-CSF generates macrophages with a transcriptomic profile similar to their unstimulated counterparts, but with a distinct chromatin accessibility landscape. Our combined transcriptome/ATAC analysis shows the potential of macrophages, among non-adherent cells in the EB myeloid differentiation phase, to be truly naïve cells early on, whereas 24 h into the macrophage differentiation phase, macrophages are reversibly activated. We also identified multiple features resembling YS myelopoiesis in our iPSC system. Nonetheless, despite the YS origin of Kupffer cells (KC)^81,84^ the LR classifier from foetal liver KC did not capture any in vitro subpopulation, indicating that the strong tissue-resident signature of KC is not recapitulated using this protocol. In particular, we did not observe activation of KC-determining TF LXRa, RBPJ or SMAD4, probably due to a lack of liver-derived signals such as Notch ligand DLL4 essential for their induction^85^. All considered, the ability to derive certain macrophage subtypes in vitro, combined with high-resolution single-cell analysis provides the unprecedented possibility to directly manipulate these cells and interrogate the extent of intrinsic macrophage plasticity, which remains a matter of debate^86^. Additionally, a marked enrichment of the macrophage population was observed during the macrophage differentiation phase, pointing to the need for single-cell approaches at distinct time points. Overall, the exhaustive analysis of this in vitro protocol will guide the applications of this system to model macrophages.

Finally, we leveraged this model to experimentally evaluate genes linked to immune-related phenotypes by GWAS. Interestingly, CRISPR/Cas9-mediated KO of GWAS hits (*PRKCB*, *LSP1* and *ICAM1*) in iPSC-derived macrophages and DCs highlighted their potential role in physiological and pathological cell states of distinct cell types. Specifically, macrophage differentiation in KO lines altered inflammatory and extracellular matrix genes. Fibrosis constitutes a pathological feature of most chronic inflammatory diseases including the ones featured in our study^87,88^, and our results open an avenue for therapeutic intervention in these disorders. In line with this, we show that both the foetal-like *FOLR2*+ and the *SPP1*+ TAM states observed in liver cancer^27^ are recapitulated in this system. This suggests these cells could also be a faithful model to unravel the role of macrophage subtypes in the tumour microenvironment.

In conclusion, we have defined a comprehensive map of cells and molecular programmes that underlie iPSC–macrophage differentiation in a dish. Macrophages play an important role for immunity in health and disease, and represent key cellular targets for immunotherapy. Our study shows the potential of deeply characterising differentiation protocols at the single-cell level, and demonstrates that these iPSC myeloid differentiation protocols are valuable models for interrogating the very early stages of hematopoietic formation that have been largely unexplored so far.

## Methods

Reagent/material details can be found in the Supplementary Information.

### Human induced pluripotent stem cell lines

All iPSC lines used in the study were generated by the HIPSCI project. Details on their generation are available at http://www.hipsci.org. Briefly, we used kolf_2, yemz_1 and vass_1 in the Discovery and DC data sets, and we added ceik_1, eesb_1 and wegi_1 for the Validation data sets. All cells in the KO data set are derived from kolf_2 as a parental line, which was subcloned prior to editing (kolf_2_C1). All HIPSCI samples were collected from consenting research volunteers recruited from the NIHR Cambridge BioResource (http://www.cambridgebioresource.org.uk), initially under existing ethics rules for iPSC derivation (Regional Ethics Committee (REC) reference 09/H0304/77, v.2, 4 January 2013), with later samples collected under a revised consent (REC reference 09/H0304/77, v.3, 15 March 2013).

### In vitro differentiation to macrophages and dendritic cells

We used an adaptation of the van Wilgenbrug et al. protocol^15^. Feeder-free human iPSC lines were cultured in E8 (StemCell Technologies) on vitronectin-coated plates (Life Technologies). For the embryoid body (EB) formation, step1, a single-cell suspension of hiPSC was plated in 100 µl of EB medium – E8 + SCF (20 ng/ml, Peprotech) + VEGF (50 ng/ml, Peprotech) + BMP-4 (50 ng/ml, Peprotech) + ROCK inhibitor (10 µM, Sigma) – at a density of 10,000 cells per well in round bottom low-attachment 96 well plates (Corning). After 2 days, we changed half the media (50 µl) and replaced it with fresh EB media. At day4, EB myeloid differentiation started, step 2, when EBs were plated in gelatin-coated 6-well plates (Sigma Aldrich) at a density of 8–10 EBs per well in EB-Mac medium – StemPro-34 (Life Technologies) + M-CSF (100 ng/ml, Peprotech) and + IL-3 (25 ng/ml, Peprotech). The EB-Mac medium was changed every 4 to 5 days. At day31, step 3, non-adherent cells were collected by centrifugation from the culture media and 1×10^6^ cells were cultured in 10 cm tissue culture plates for 7 days in macrophage differentiation medium – RPMI (ThermoFisher) + 10% heat-inactivated FBS + M-CSF (100 ng/ml, ThermoFisher).

Alternative macrophage differentiation media were used in the macrophage differentiation phase, step 3. For the cytokines experiment (Fig. 3b–d), we used RPMI + 10% heat-inactivated FBS + GM-CSF (50 ng/ml, Peprotech) and RPMI + 10% heat-inactivated FBS + GM-CSF (10 ng/ml) + IL-34 (100 ng/ml, Peprotech), for the latter cells were plated at 6×10^5^ cells per well of a 6well plate. For the media experiment (Fig. 3b and Supplementary Fig. 7A), fully defined medium – StemPro-34 + M-CSF (100 ng/ml) - was used.

Step 1 is shared between macrophages and DCs, while different cytokines are used in steps 2 and 3. For DC differentiation EBs at day4 are plated with EB-DC media – StemPro-34 + GM-CSF (50 ng/ml) + FLT3L (100 ng/ml, Peprotech) in the same types of plates and density as the macrophage protocol. At day31, step 3, non-adherent cells were collected and plated in 10 cm tissue culture plates in DC differentiation medium – RPMI + 10% heat-inactivated FBS + GM-CSF (50 ng/ml) + IL-4 (100 ng/ml, Peprotech).

### LPS stimulation of iPSC-derived cells

Four populations of cells derived from the macrophage protocol described above were stimulated with LPS (i.e. non-adherent cells from the EB myeloid differentiation phase -Mac Day0-, cells at the end of the Macrophage differentiation phase incubated for 7 days with M-CSF (100 ng/ml) -Mac Day7 M-CSF-, with GM-CSF (50 ng/ml) -Mac Day7 GM-CSF- and with GM-CSF (10 ng/ml) + IL-34 (100 ng/ml) -Mac Day7 GM-CSF IL-34-). For each sample, LPS (Sigma Aldrich) was added to the media in two wells for a final concentration of 2.5 ng/ml while a third well was kept as control. After 2 h, Brefeldin A (Sigma Aldrich) was added to reach 5ug/ml to all wells and cells were incubated for an additional 4 h (total 6 h of stimulation with LPS). Media was collected for control and LPS wells for cytokine analysis using V-PLEX Proinflammatory Panel 1 Human Kit (Mesoscale) according to manufacturing instructions. In parallel, cells were collected using 10 mg/ml Lidocaine (Sigma Aldrich) 2 mM EDTA (ThermoFisher) solution for 5 min at 37 °C, the two wells of LPS stimulation per population were combined. Single-cell RNAseq analysis was performed aiming at 3000 cells per condition as described in ‘10× Genomics Chromium GEMs sample preparation and sequencing’.

### 10× Genomics Chromium GEM sample preparation and sequencing

Single-cell transcriptomic analysis on iPSC-to-macrophage differentiation was performed in 3 iPSC lines for the Discovery data set and 6 hiPSC lines for the Validation data set. One 6-well well per line was collected using TrypLE (Life Technologies) at 20 time points in the Discovery data set, and 2 6-well wells per line at 7 time points in the Validation data set, between day0 and day38 (day31 EBs +7 days of the macrophage differentiation phase). At every collection day, all cells from the wells or plates were detached, merged, counted, passed through 40 µM filters and resuspended in DPBS (ThermoFisher) + 0.4% BSA (GE Healthcare). At the day31 collection day, we additionally collected sample ‘Day31 non-adh’ which corresponds to the non-adherent fraction of the culture, it was processed as the rest but no detachment step was necessary. Cell suspensions were processed using the Chromium Single Cell 3’ kit (v2 for Discovery, v3 for Validation, 10X Genomics), aiming at recovering from 3000 to 10000 cells. Library preparation was carried out according to the manufacturer’s instructions. Libraries were sequenced, aiming at a minimum coverage of 50000 raw reads per cell, on the Illumina HiSeq 4000 (Discovery) or Novaseq 6000 (Validation) using the sequencing formats; read 1: 26 cycles; i7 index: 8 cycles, i5 index: 0 cycles; read 2: 98 cycles (3’ kit v2) or read 1: 28 cycles; i7 index: 8 cycles, i5 index: 0 cycles; read 2: 91 cycles (3’ kit v3).

Sample preparation and sequencing for the DC data sets were performed as described for the Discovery data set. The Knockout data set samples were processed as described for the validation data set but only for 2 time points (i.e. day0 and day31).

Single-cell ATAC analysis was performed in a subset of the single-cell suspensions for 6 of the time points of the Validation data set described above. Single-nuclei suspensions were obtained and processed according to the manufacturer’s instructions using Chromium Single Cell ATAC v1.0 (10X Genomics), aiming for 10000 nuclei per sample. Library preparation was carried out according to the manufacturer’s protocol and sequenced on Illumina NovaSeq 6000, aiming for 20000 fragments per cell using the sequencing formats; read 1: 50 cycles; i7 index: 8 cycles, i5 index:16 cycles; read 2: 50 cycles.

### Single-cell RNA seq computational analysis

Cell Ranger (v3.1.0), mapping to GRCh38 (v3.0.0), was used to filter out empty droplets using default values. Cells were further filtered out for the number of genes (<200) and percentage of mitochondrial RNA (>8.5%) using Seurat (https://satijalab.org/seurat/ v3.2.2), R v4.0. All cells identified as doublets using SoupOrCell^89^, DoubletDetection (10.5281/zenodo.2678041) and Scrublet^90^ were discarded. Cell genotype calling was performed using SoupOrCell^89^. All data sets were normalised using sctransform in Seurat^91^, and UMI counts, mitochondrial RNA and cell cycle variables were regressed out by cell line. Multiple hiPSC lines were integrated using Seurat’s anchor-based method^24^. After PCA dimensionality reduction and louvain clustering^92^, data sets were further analysed as described below.

### Single-cell ATAC seq computational analysis

Cell Ranger ATAC pipeline (v1.2.0), mapping to GRCh38 (v3.0.0), was used for read filtering and barcode cell calling. Peaks were re-called using cellatac, an in-house implementation of Cusanovich’s approach^93^ (https://github.com/cellgeni/cellatac)^94^. Peak and cell filtering were performed using cellatac and Signac (https://satijalab.org/signac/ v1.1.1), as described in Supplementary Fig. 1B. Normalisation and dimensionality reduction were performed using term frequency-inverse document frequency (TF-IDF) and Singular Value Decomposition (SVD), respectively. SLM from Seurat was used for clustering. TF motif analysis was performed using Signac and JASPAR 2020^95^ motifs database.

### Cell-type annotation of RNA and ATAC data sets

Both the Discovery and DC data sets were annotated using logistic regression (LR) models built on publicly available single-cell transcriptomic data sets. The LR prediction models used at each step were built based on a general linear model function and a tenfold cross-validation. Briefly, public raw data (Cell Ranger output when available or processed matrices otherwise) was downloaded and re-processed as described for the Discovery and DC data sets. Then, public data sets were split into training (70%) and test (30%) sets, ensuring these proportions were accounted for each cell type. Then we generated LR models to classify each cell type for each gene in the training partition of the in vivo data set using normalised data. A ranked gene list based on the area under the curve (AUC) of each gene was produced for each cell type. The optimal number of genes to build the final LR classifier was chosen by building models on the training set and calculating the AUC of the prediction on the test set. This was repeated with an increasing number of genes down the ranked list described above. The number of genes that produced a model on the training set with the highest AUC when applied to the test set was then used to build the final model on the full in vivo data set. This LR prediction model was then used to classify the cells in the in vitro data set. Finally, the mean prediction probability per louvain cell cluster was calculated for all the LR models built, and each cluster was labelled based on the LR model with the highest mean prediction. As an estimate of the strength of the association between the annotation and the labelled clusters, we calculated the AUC of each annotated cell type based on the LR probabilities.

For the validation data set, cell type annotations from the Discovery data set were projected on the transcriptomic and ATAC validation data sets using Seurat’s anchor-based label transfer approach^24^.

### Trajectories analysis

Spliced/unspliced RNA expression matrices were generated using the command line tool from velocyto (http://velocyto.org/velocyto.py/tutorial) on Python v3.8. scVelo was used for trajectory analysis based on RNA velocity and PAGA graph abstraction as described (https://scvelo.readthedocs.io/DynamicalModeling/ v0.2.2) on Python v3.8. All analyses were performed on a per sample basis. Progenitor and derivative cell type associations, shown in Fig. 2 a, were the most prevalent connections observed in the scVelo analysis across all samples analysed. Additionally, the proposed progenitors shown were observed earlier or simultaneously to the derivative cell type along the time series. If the direction inferred with the scVelo analysis contradicted the order of appearance of the potential progenitor and derivative cell types, the association was not considered.

### Transcription factor activity analysis

Transcriptomic changes across trajectories and time points were studied based on transcription factor activities using DoRothEA and VIPER analysis^30^. DoRothEA v1.2.1 (https://saezlab.github.io/dorothea) required Seurat v4.0.2 (https://satijalab.org/seurat/). Both in vitro and in vivo data sets were subset based on connected cell types according to the trajectory analysis. Normalised data was scaled within each subset, and TF activity scores were computed for each cell for 271 TFs with high-confidence target-gene annotation (A, B and C confidence levels, https://saezlab.github.io/dorothea/). Heatmaps for in vitro vs in vivo comparison were produced by selecting the top 50 most variable TFs in each data set, and results were merged and plotted using pheatmap (https://www.rdocumentation.org/packages/pheatmap/versions/1.0.12). Significance analysis was performed with FindMarkers function from Seurat (https://satijalab.org/seurat/, v4.0.2) using the LR method, *p* values were adjusted using Bonferroni correction.

### Marker protein and antigen processing DQ-OVA assay analysis by FACS

Macrophages and dendritic cells were detached from 10 cm plates using 10 mg/ml Lidocaine 2 mM EDTA solution for 5 min at 37 °C, collected in DPBS and spun down at 300 × *g* for 3 min. Samples were then fixed with BD Cytofix/Cytoperm buffers (ThermoFisher) for 20 min at room temperature and washed with DPBS + 1% FBS. Staining with fluorescent-labelled primary antibodies (see Supplementary Information for antibody details) was performed in the dark at room temperature for 30 min. After 2 washes with DPBS + 1% FBS, cells were analysed by FACS in a BD LSR Fortessa II and data was analysed using FlowJo v10. The gating strategy is provided in Supplementary Fig. 4B.

Dendritic cells were collected as described above and incubated with DQ-OVA (ThermoFisher) in the dark at 4 °C or 37 °C for 15 min, 45 min and 60 min, as indicated. Cells were then washed with ice-cold DPBS + 1% FBS and analysed by FACS as above.

### T cell activation assay by CFSE staining and FACS

Twenty thousand iPSC-derived non-adherent cells from the macrophage or DC differentiation protocol were differentiated to DC (in presence of GM-CSF and IL-4) or macrophages (in the presence of M-CSF) for 7 days. This was performed as described for the last phase of differentiation in the protocols used throughout the study but in round-bottom 96 well plates. In parallel, peripheral blood mononuclear cells (PBMCs) were isolated using Ficoll-Paque PLUS (GE Healthcare) density gradient centrifugation. Naïve T cells were isolated from the PBMC fraction using EasySep® human naïve CD4 + T cell isolation kits II (StemCell Technologies) according to the manufacturer’s instructions. After the 7 days of DC and macrophage differentiation described above, 100.000 naïve T cells, stained with CFSE (ThermoFisher), were stimulated with anti-CD3 (Tonbo Biosciences, 70-0037-U100, 100 ng/1million cells) in the presence of iPSC-derived dendritic cells, iPSC-derived macrophages or anti-CD28/CD3 (StemCell Technologies, 10971, 6ul/1million cells). After 4 days of co-culture, T cell proliferation was measured by assessing CFSE dilution. Anti-CD3 alone was tested independently and had no effect on T cell proliferation rate. The experiment was performed in two independent experiments using two PBMC donors each time in one of the iPSC lines (kolf_2). The gating strategy is provided in Supplementary Fig. 9G.

### CRISPR-Cas9 KO of human induced pluripotent stem cell lines

KO iPSC lines were generated by substituting an asymmetrical exon with a Puromycin cassette and expanding those clones with a frame-shift indel in the remaining allele. A hSpCas9 and two small guide RNA expression vectors along with a homology-directed repair template vector were used. The template vector harboured an EF1a-Puromycin cassette with two flanking 1.5 kb homology arms designed around the asymmetric exon of interest. For each KO line, 2 × 10^6^ iPSC single cells were transfected using the Human Stem Cell Nucleofector® Kit 2 (Lonza) with 4 μg, 3 μg and 2 μg of each plasmid, respectively, and plated in 10 cm plates. After 72 h, cells were selected in 3 μg/mL Puromycin (Invivogen) and colonies were expanded and genotyped. Lines confirmed to have the Puromycin cassette and the presence of a frame-shift indel by Sanger sequencing (Supplementary Information) were selected for the experiments.

### KO iPSC-derived cell types analysis

Transcriptomic alterations between cell types arising in WT and KO lines were assessed using differential expression from Seurat. Genes present in 10% of the cells and with a minimal log fold change of 0.25 were selected for differential expression analysis of each cell type in each KO line vs their WT counterpart. Only genes with an FDR < 0.05 were considered as significantly differentially expressed. Differential amount of cells produced was calculated using two-sided *t* test between *ZEB2* KO number of cells collected for the two clones used vs the number of cells collected for all other lines.

### Reporting summary

Further information on research design is available in the Nature Research Reporting Summary linked to this article.

## Supplementary information

Supplementary Information

Description of Additional Supplementary Files

Supplementary Data

Reporting Summary

## Data Availability

The single-cell sequencing fastq files are available in the ArrayExpress database (http://www.ebi.ac.uk/arrayexpress) under accession numbers E-MTAB-11623 for single-cell RNAseq and E-MTAB-11616 for single-cell ATACseq. Processed data sets can be queried and downloaded through the web portal www.HiPImmuneatlas.org. Single-cell sequencing data was mapped against GRCh38 http://ftp.ensembl.org/pub/release-100/. Publicly available data sets used include Gastrulation http://www.human-gastrula.net/, Foetal liver (+kidney +skin) E-MTAB-7407, Foetal thymus E-MTAB-8581, Placenta E-MTAB-6701, HCC 10.17632/6wmzcskt6k.1, Yolk sac GSE133345 and adult DCs and Macs GSE115006. Source data are provided with this paper.

## Source data

Source Data

## Code availability

All relevant codes used for data analysis are available from https://github.com/Ventolab/iPSCmyeloid.
