# Supplementary material for: Robust temporal map of human in vitro myelopoiesis using single-cell genomics
Source: Nat Commun. 2022 May 24;13:2885. doi: 10.1038/s41467-022-30557-4 (PMC9130280; doi:10.1038/s41467-022-30557-4)
Supplement: Supplementary file 1 — Supplementary Information [file 41467_2022_30557_MOESM1_ESM.pdf]

SUPPLEMENTARY INFORMATION for the study:

Robust temporal map of human *in vitro* myelopoiesis using single-cell genomics

Clara Alsinet1,2*, Maria Nascimento Primo2,3, Valentina Lorenzi3, Erica Bello2,3, Iva Kelava3, Carla P Jones3, Roser Vilarrasa-Blasi3, Carmen Sancho-Serra3, Andrew J Knights3, Jong-Eun Park4, Beata S Wyspianska2,5, Gosia Trynka2,3, David F Tough5, Andrew Bassett2,3, Daniel J Gaffney6*, Damiana Alvarez-Errico7*, Roser Vento-Tormo8*

1. Wellcome Sanger Institute, Wellcome Genome Campus, Hinxton, Cambridge CB10 1SA, UK.
2. Open Targets, Wellcome Genome Campus, Hinxton, Cambridge CB10 1SA, UK.
3. Wellcome Sanger Institute, Wellcome Genome Campus, Hinxton, Cambridge CB10 1SA, UK.
4. Graduate School of Medical Science and Engineering, Korea Advanced Institute of Science and Technology (KAIST), Daejeon 34141, Korea.
5. Immunology Research Unit, Medicines Research Centre, GlaxoSmithKline, Stevenage SG1 2NY, UK.
6. Wellcome Sanger Institute, Wellcome Genome Campus, Hinxton, Cambridge CB10 1SA, UK.
7. Josep Carreras Leukaemia Research Institute (IJC), 08916, Badalona, Barcelona, Catalonia, Spain.
8. Wellcome Sanger Institute, Wellcome Genome Campus, Hinxton, Cambridge CB10 1SA, UK.

*co-corresponding authors

Contents:

Supplementary Figures

- Supplementary Figure 1. Computational workflow
- Supplementary Figure 2. Logistic regression predictions from in vivo datasets for cell types in the Discovery dataset
- Supplementary Figure 3. Progression of cell types throughout the differentiation protocol time points
- Supplementary Figure 4. FACS analysis of the presence of differentiated Mast cells
- Supplementary Figure 5. Further characterisation of the Discovery dataset
- Supplementary Figure 6. Additional supporting data for the trajectories analysis
- Supplementary Figure 7. Additional supporting data for the macrophage differentiation phase
- Supplementary Figure 8. Logistic regression predictions from in vivo datasets for cell types in the DC dataset
- Supplementary Figure 9. Dendritic cells protocol
- Supplementary Figure 10. Sanger Sequencing results from KO lines
- Supplementary Figure 11. Knock-out cell types and transcriptomic profiles

Supplementary Methods

- Reagent/Materials table. Detailed information about all products mentioned on the Methods section
- Antibodies table. Detailed information about all antibodies used including clone, lot and dilution
- Primers table. Primer sequences for sanger sequencing of human iPSC knock out loci

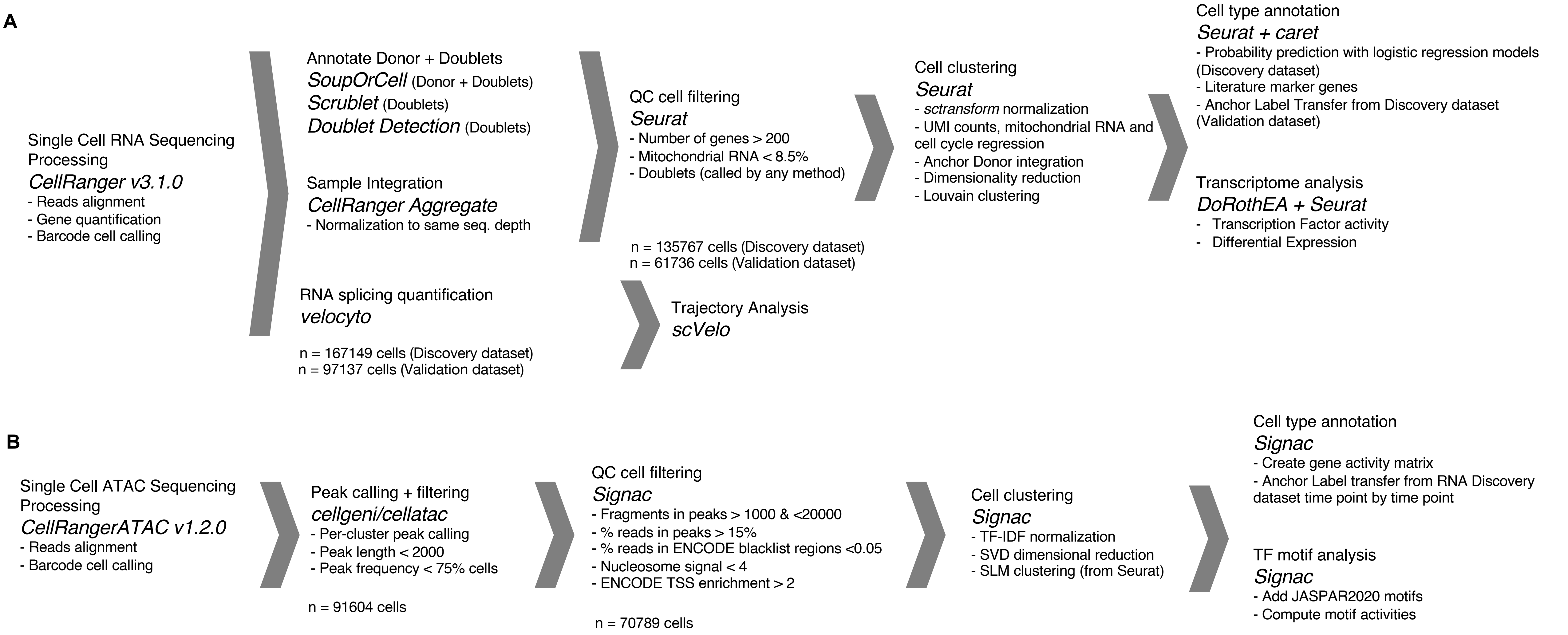

Supplementary Figure 1. Computational workflow

A, Computational processing and analysis of 10X Genomics Chromium single-cell GEMs 3' RNA samples. **B,** Computational processing and analysis of 10X Genomics Chromium single-nuclei GEMs ATAC samples.

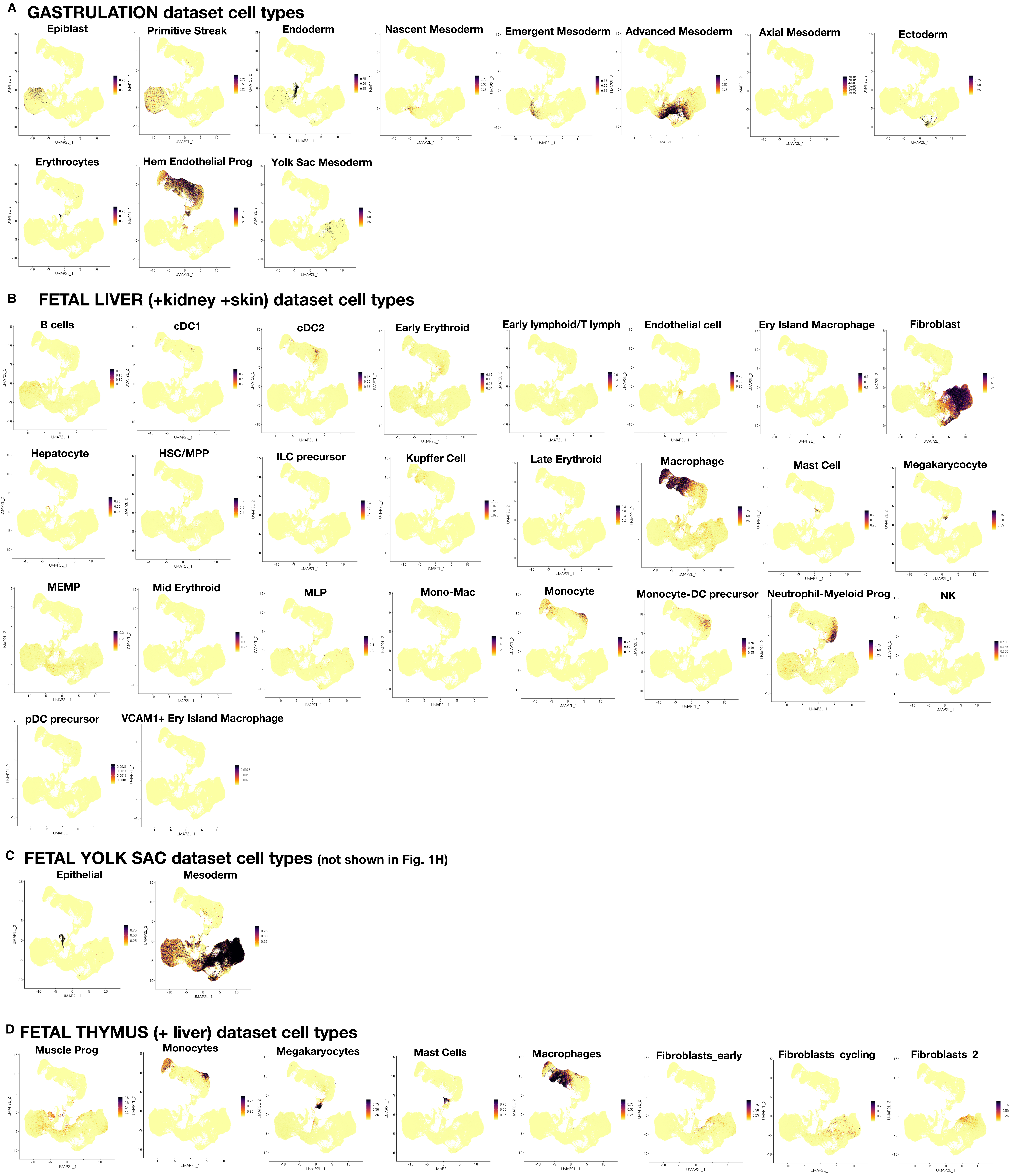
A GASTRULATION dataset cell types
Epiblast
Primitive Streak
Endoderm
Nascent Mesoderm
Emergent Mesoderm
Advanced Mesoderm
Axial Mesoderm
Ectoderm
Erythrocytes
Hem Endothelial Prog
Yolk Sac Mesoderm
B FETAL LIVER (+kidney +skin) dataset cell types
B cells
cDC1
cDC2
Early Erythroid
Early lymphoid/T lymph
Endothelial cell
Ery Island Macrophage
Fibroblast
Hepatocyte
HSC/MPP
ILC precursor
Kupffer Cell
Late Erythroid
Macrophage
Mast Cell
Megakaryocyte
MEMP
Mid Erythroid
MLP
Mono-Mac
Monocyte
Monocyte-DC precursor
Neutrophil-Myeloid Prog
NK
pDC precursor
VCAM1+ Ery Island Macrophage
C FETAL YOLK SAC dataset cell types (not shown in Fig. 1H)
Epithelial
Mesoderm
D FETAL THYMUS (+ liver) dataset cell types
Muscle Prog
Monocytes
Megakaryocytes
Mast Cells
Macrophages
Fibroblasts_early
Fibroblasts_cycling
Fibroblasts_2
UMAP2L_1
UMAP2L_2

CONTINUED from above

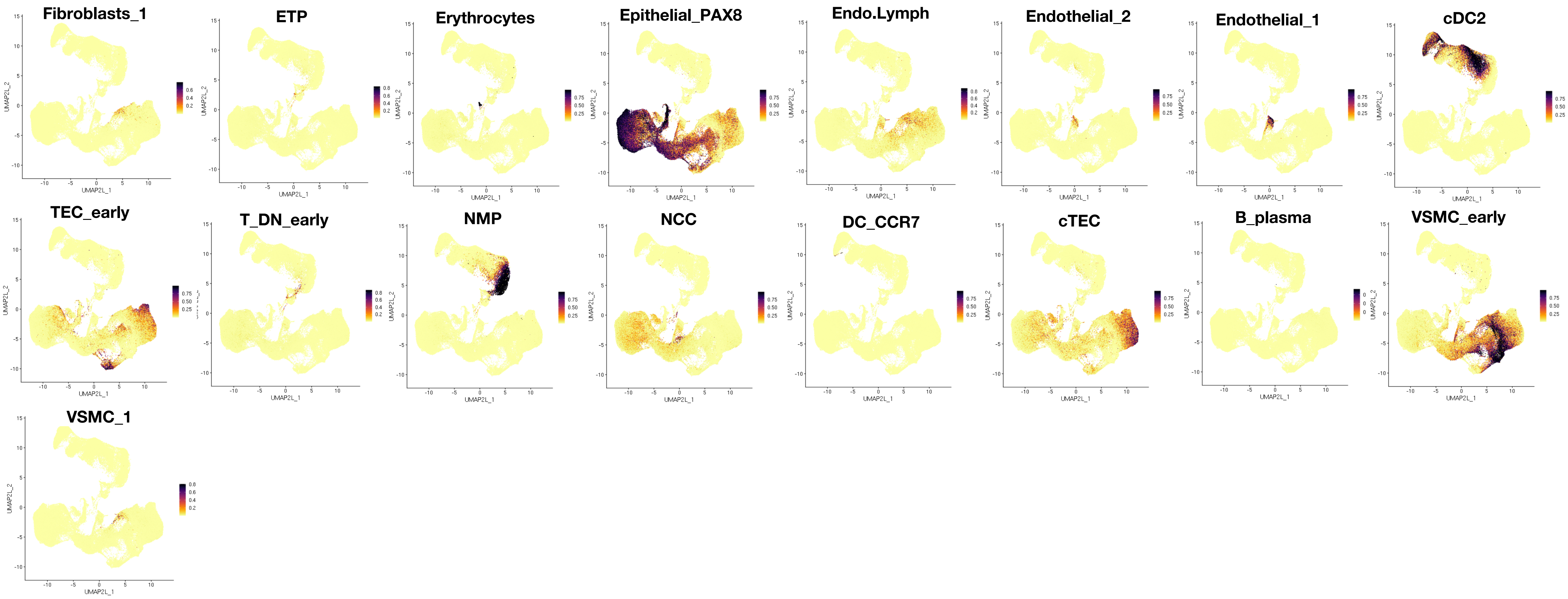

E **PLACENTA dataset cell types**

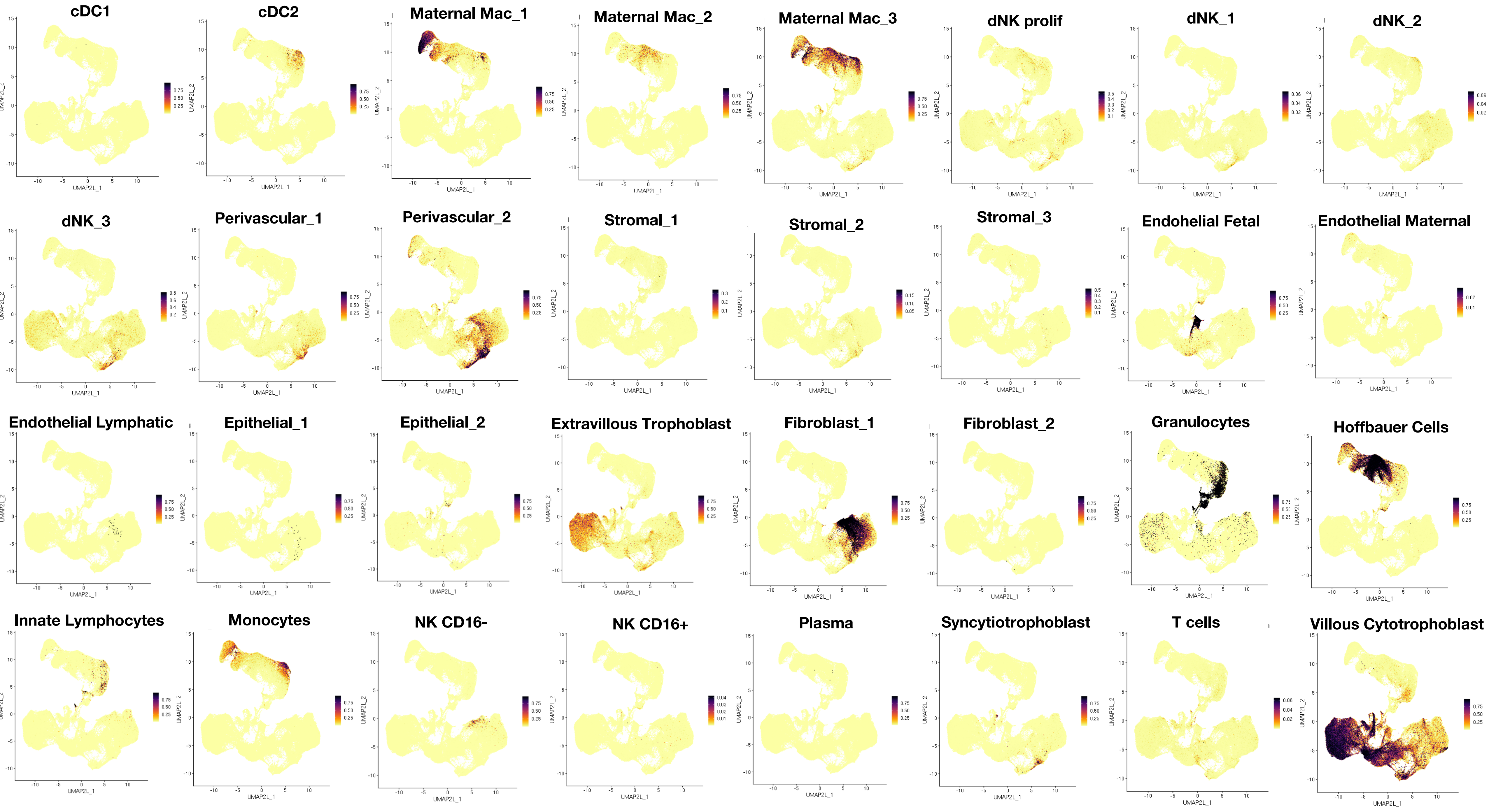

Supplementary Figure 2. Logistic regression predictions from *in vivo* datasets for cell types in the Discovery dataset
Discovery dataset UMAP projections showing the logistic regression prediction probabilities for models trained on each cell type present in publicly available single-cell transcriptomic datasets. Prediction probabilities built on: **A,** Human gastrulation embryo dataset, 2-3 post conceptional weeks (PCW)[23], **B,** Human fetal liver, skin and kidney cells, 7-17 PCW[22], **C,** Human fetal yolk sac 4-5 PCW[6], **D,** Human fetal thymus and liver cells, 7-17 PCW[21] and, **E,** Human fetal, 6-12 PCW, and decidual, adult, cells[20].

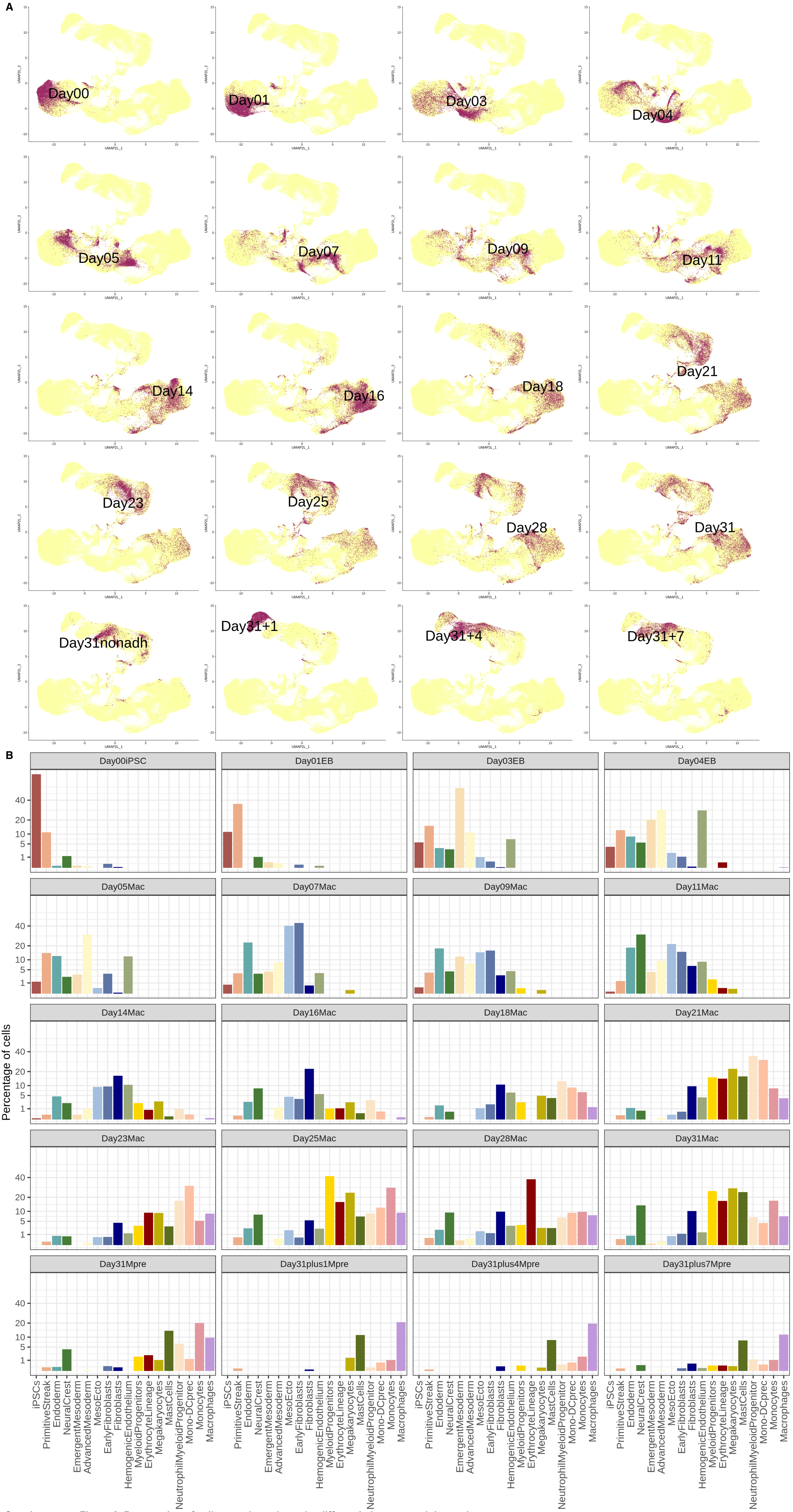

Supplementary Figure 3. Progression of cell types throughout the differentiation protocol time points

A, Discovery dataset UMAP projections where cells corresponding to each of the time points / samples are highlighted. **B,** Barplots showing the proportion of cells for each cell type across all time points / samples analysed in the Discovery dataset.

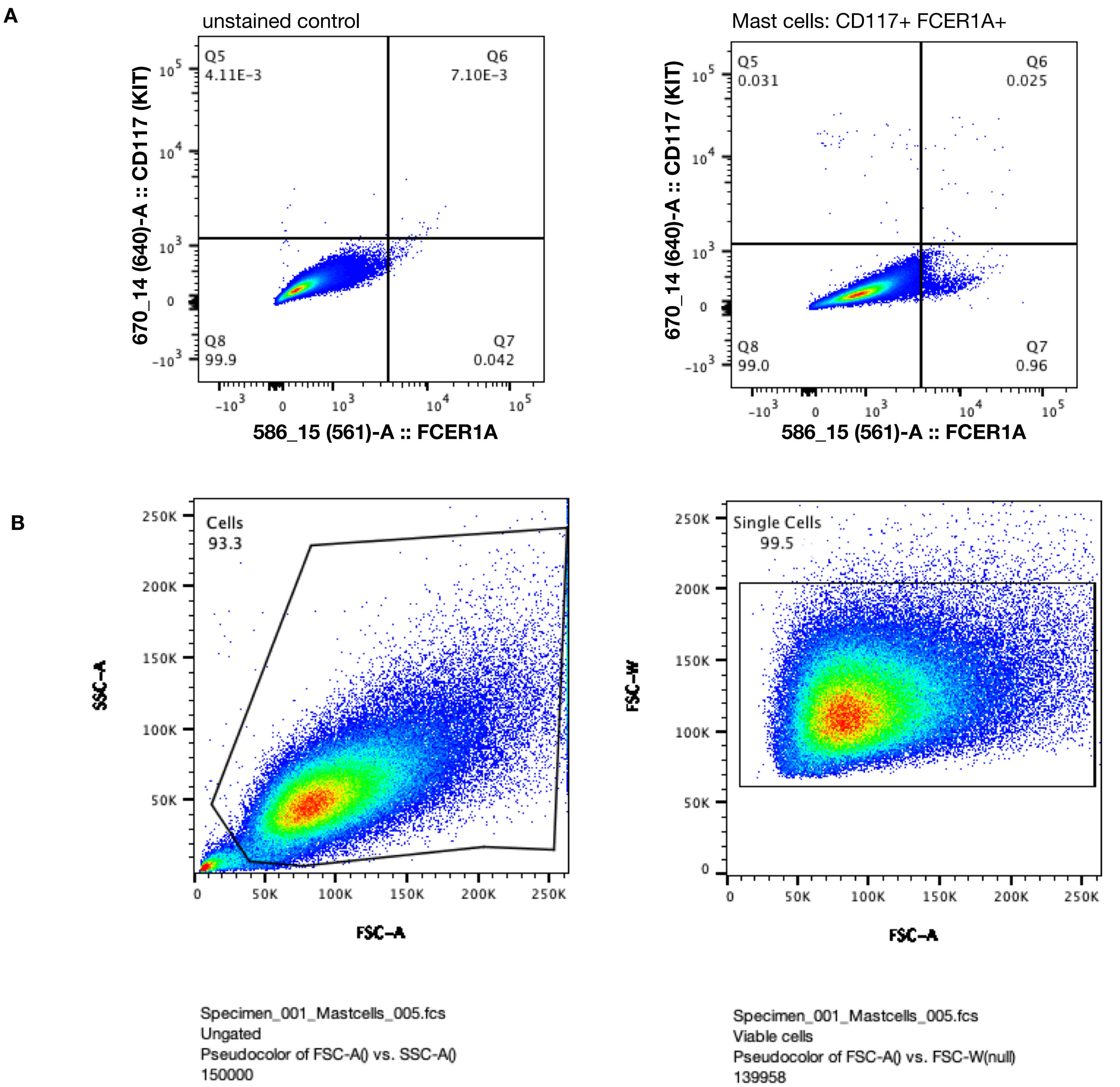

Supplementary Figure 4. FACS analysis of the presence of differentiated Mast cells

A, FACS plots showing CD117 (KIT) and FCER1A mast cell markers protein expression levels for the non-adherent cell fraction of the EB myeloid differentiation phase for one donor (kolf_2). Plots shown are representative of three collections and analyses performed on cultures >day21, the % of CD117+ FCER1A+ cells was < 0.1% in all 3 analyses. **B,** Representative gating strategy for all FACS results of cell type markers expression.

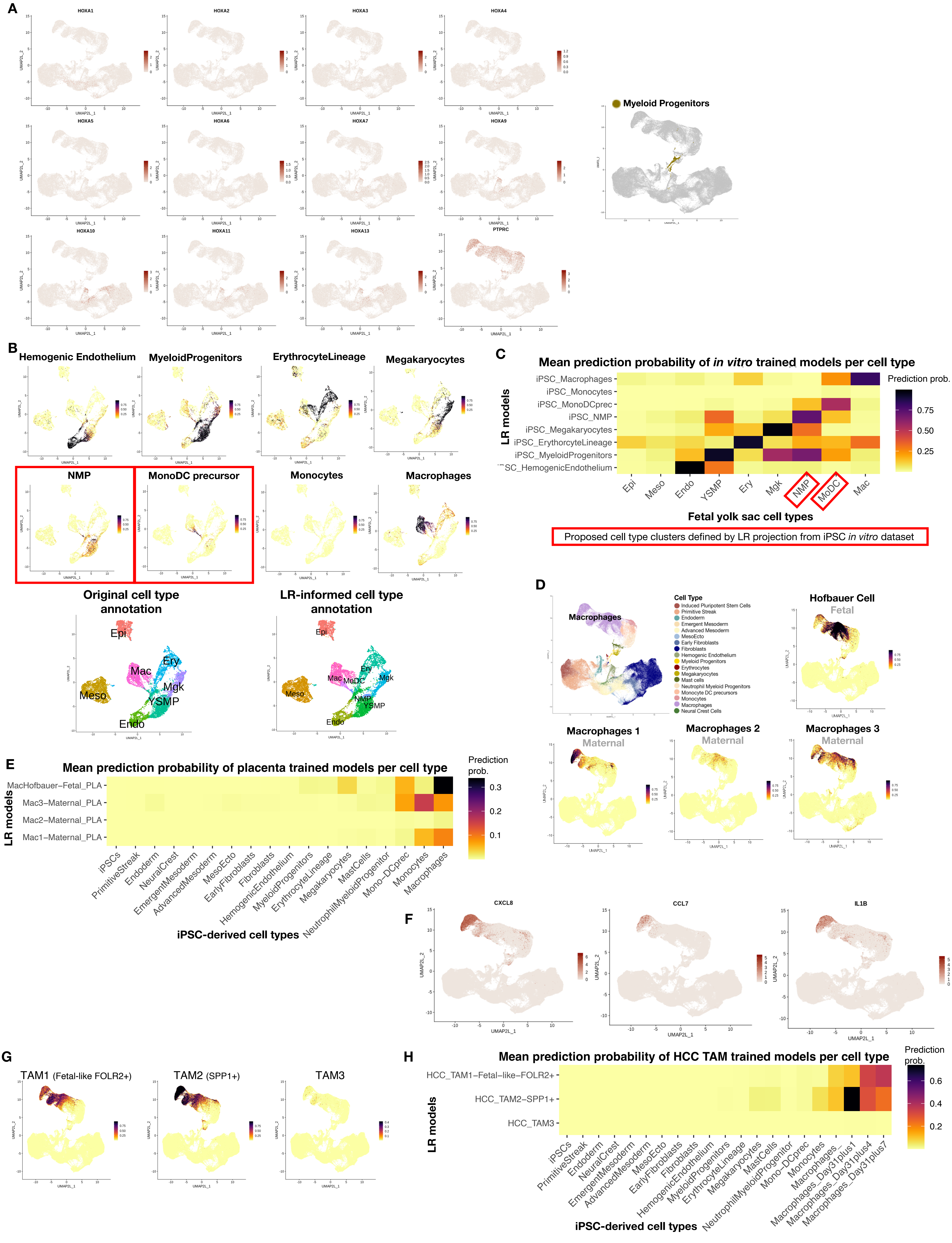

Supplementary Figure 5. Further characterisation of the Discovery dataset

A, Discovery dataset UMAP projections showing scaled gene expression for the HOXA family of genes and PTPRC. (right) UMAP projections highlighting the location of cells annotated as myeloid progenitors. **B,** UMAP projections of the yolk sac dataset[6] coloured by the predicted probabilities by logistic regression of myelopoiesis cell types from the *in vitro* iPSC-derived Discovery dataset, (bottom) UMAP projections of the cell types described in the yolk sac study[6] and cell types identified through logistic regression analysis. **C.** Heatmap showing the mean predicted probabilities by logistic regression of the cell types found in the yolk sac[6]. In red are cell type clusters not described in the original yolk sac study[6] and defined by the logistic regression results in B. **D,** Discovery dataset UMAP projections, first with the cell type annotations as reference and the rest are coloured by the predicted probabilities by logistic regression of each macrophage subtype found in the maternal–fetal interface[20]. **E,** Heatmap showing the mean predicted probabilities by logistic regression of the macrophage subtypes found in the maternal–fetal interface[20] for each of the cell types in the Discovery scRNAseq dataset. **F,** Discovery dataset UMAP projections showing the expression levels of cytokines overexpressed in the activated macrophages (day31+1 sample). **G**, Discovery dataset UMAP projections showing the logistic regression prediction probabilities trained on three subtypes of tumor associated macrophages (TAMs). **H,** Heatmap showing the mean predicted probabilities by logistic regression of three subtypes of TAMs for each of the cell types in the Discovery dataset.

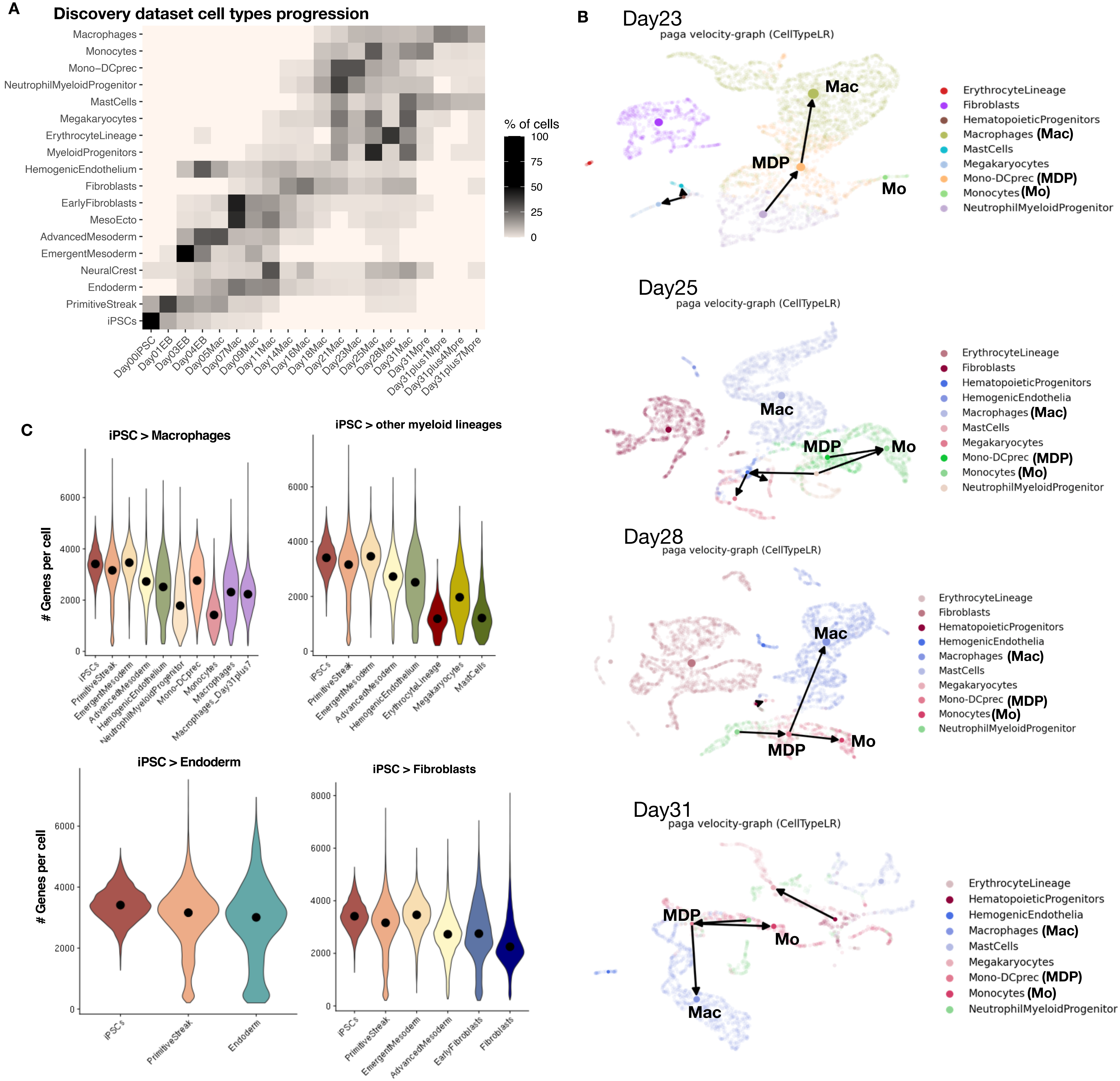

Supplementary Figure 6. Additional supporting data for the trajectories analysis

A, Heatmap of the percentage of cells distribution across all timepoints for each cell type in the Discovery dataset. **B,** Violin plots of the mean number of genes expressed per cell in each of the cell types across the main differentiation trajectories identified. Black dot = median number of expressed genes per cell type. **C**, RNA velocity analysis and PAGA graph abstraction of the cells present at day23 to 31 (EB myeloid differentiation) of the differentiation protocol (Discovery dataset) showing the developmental relationships between the myeloid cell types. Monocyte DC precursors (MDP), Monocytes (Mo) and Macrophages (Mac) are labelled on the plots.

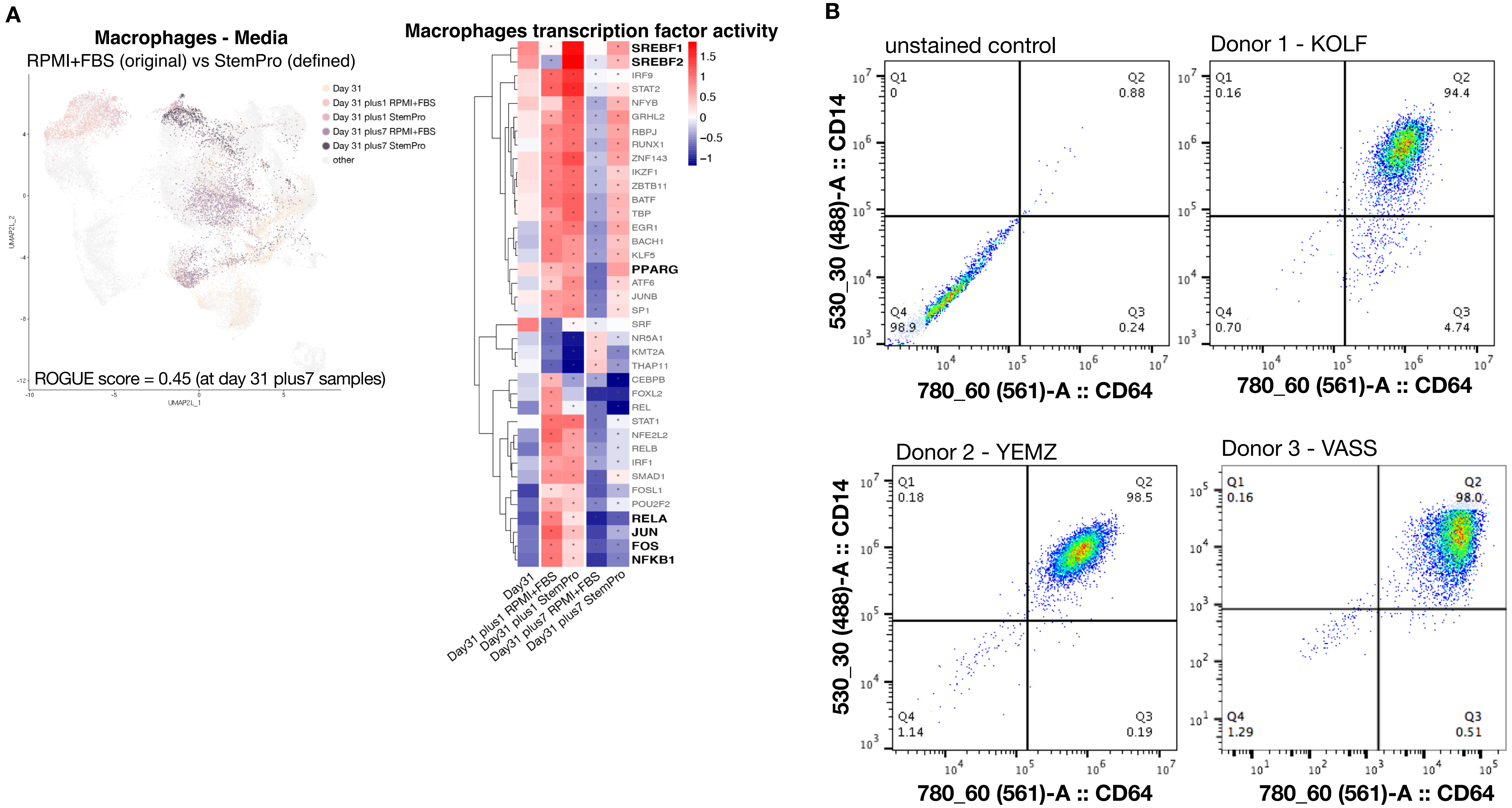

Supplementary Figure 7. Additional supporting data for the macrophage differentiation phase

A, (right) Macrophage phase UMAP projection highlighting macrophages from the media experiment and colored by time point and media composition. (left) Heatmap of the transcription factor activity scores calculated using DoRothEA across timepoints and media composition. Asterisks highlight significantly different activity vs previous time point, bonferroni adjusted p<0.05. **B,** FACS plots showing CD14 and CD64 marker protein expression levels for cells collected at the end of the macrophage differentiation phase for the three donor iPSC lines used in the discovery dataset.

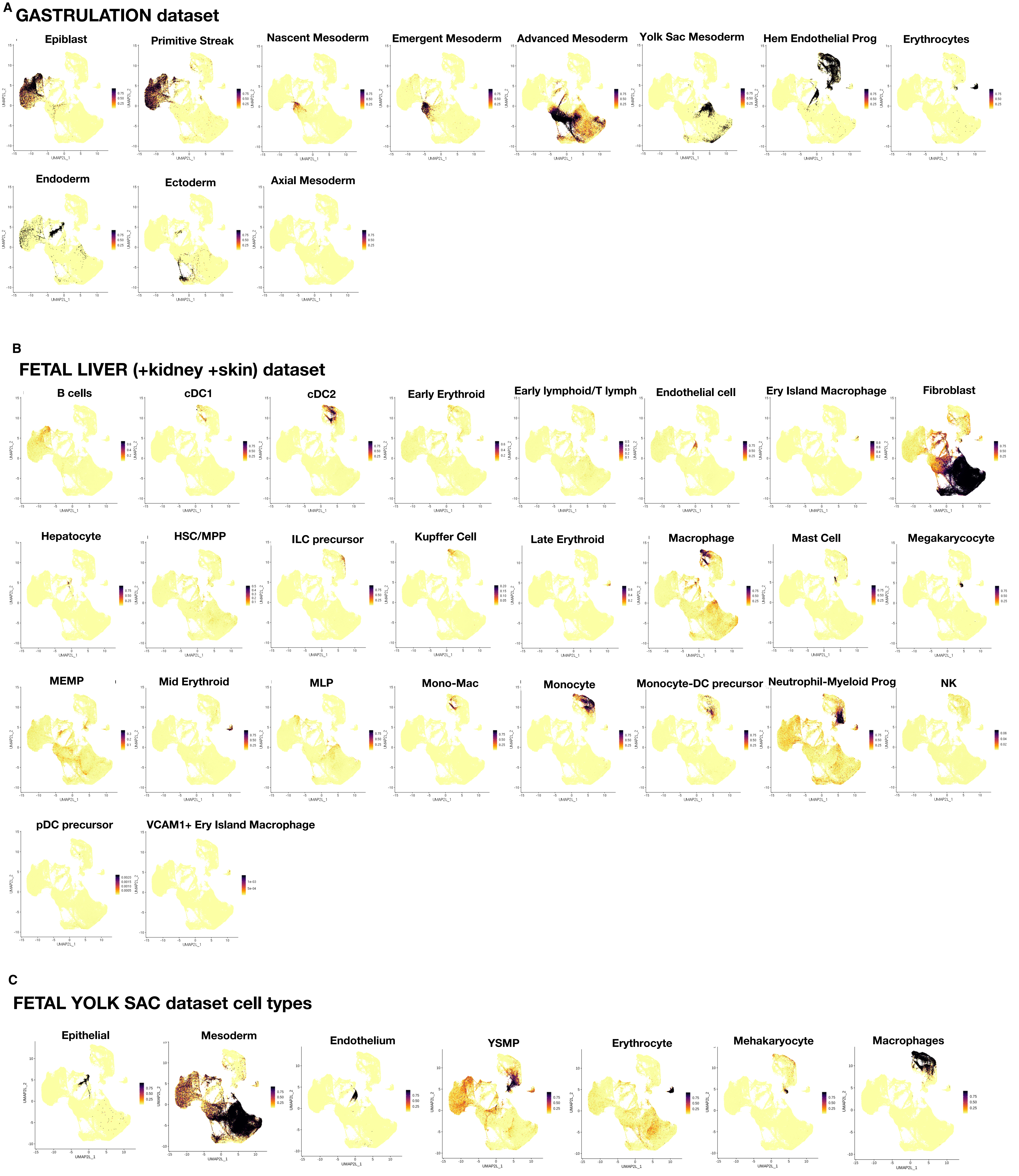
A
GASTRULATION dataset
Epiblast
Primitive Streak
Nascent Mesoderm
Emergent Mesoderm
Advanced Mesoderm
Yolk Sac Mesoderm
Hem Endothelial Prog
Erythrocytes
Endoderm
Ectoderm
Axial Mesoderm
B
FETAL LIVER (+kidney +skin) dataset
B cells
cDC1
cDC2
Early Erythroid
Early lymphoid/T lymph
Endothelial cell
Ery Island Macrophage
Fibroblast
Hepatocyte
HSC/MPP
ILC precursor
Kupffer Cell
Late Erythroid
Macrophage
Mast Cell
Megakarycocyte
MEMP
Mid Erythroid
MLP
Mono-Mac
Monocyte
Monocyte-DC precursor
Neutrophil-Myeloid Prog
NK
pDC precursor
VCAM1+ Ery Island Macrophage
C
FETAL YOLK SAC dataset cell types
Epithelial
Mesoderm
Endothelium
YSMP
Erythrocyte
Mehakaryocyte
Macrophages

CONTINUED from above

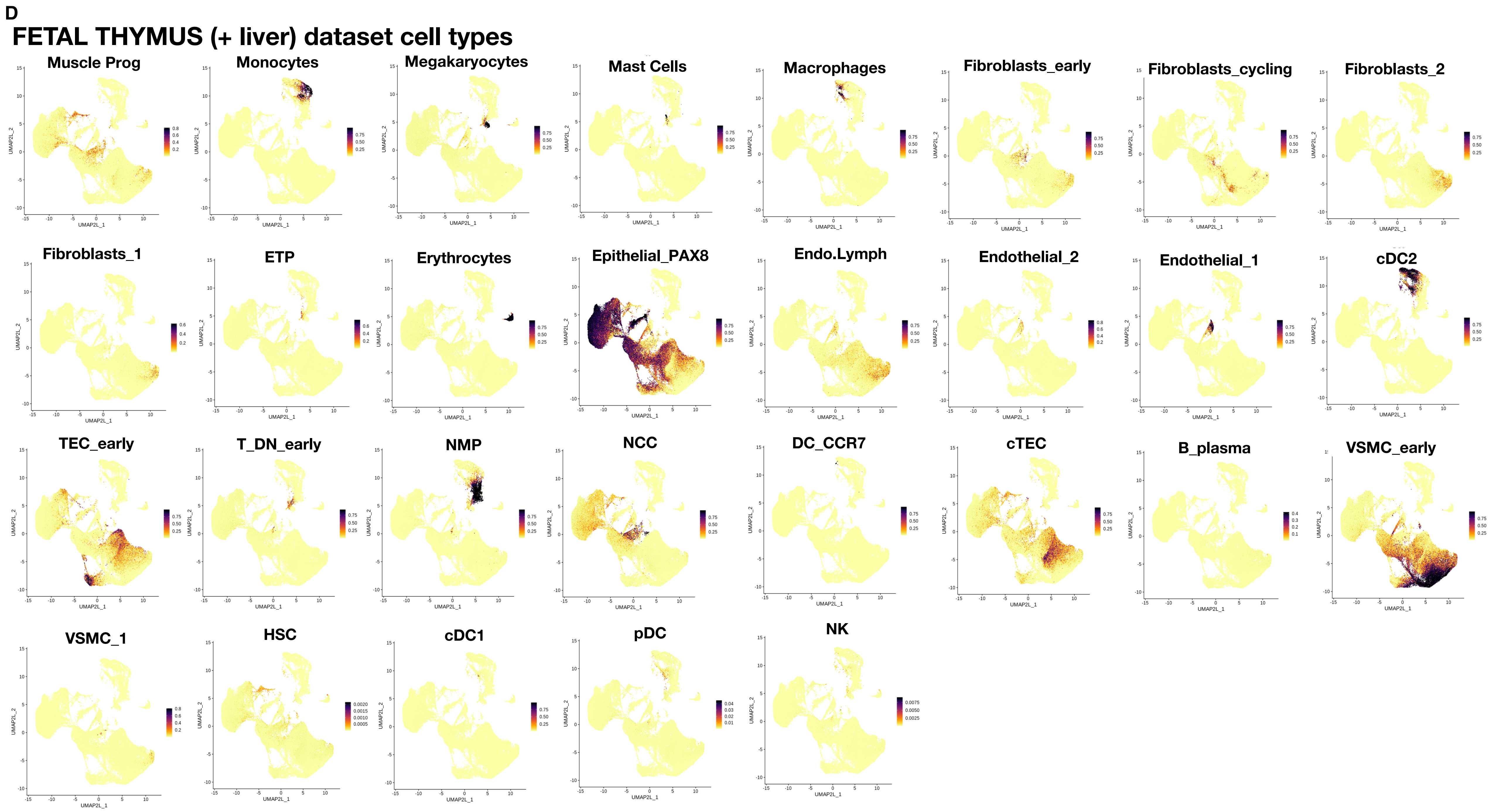

Supplementary Figure 8. Logistic regression predictions from *in vivo* datasets for cell types in the DC dataset

Dendritic cell dataset UMAP projections showing the logistic regression prediction probabilities for models trained on each cell type present in publicly available single-cell transcriptomic datasets. Prediction probabilities built on: **A,** Human gastrulation embryo dataset, 2-3 post conceptional weeks (PCW)[23], **B,** Human fetal liver, skin and kidney cells, 7-17 PCW[22], C, Human fetal yolk sac, 4-5 PCW[6] and **D,** Human fetal thymus and liver cells, 7-17 PCW[21].

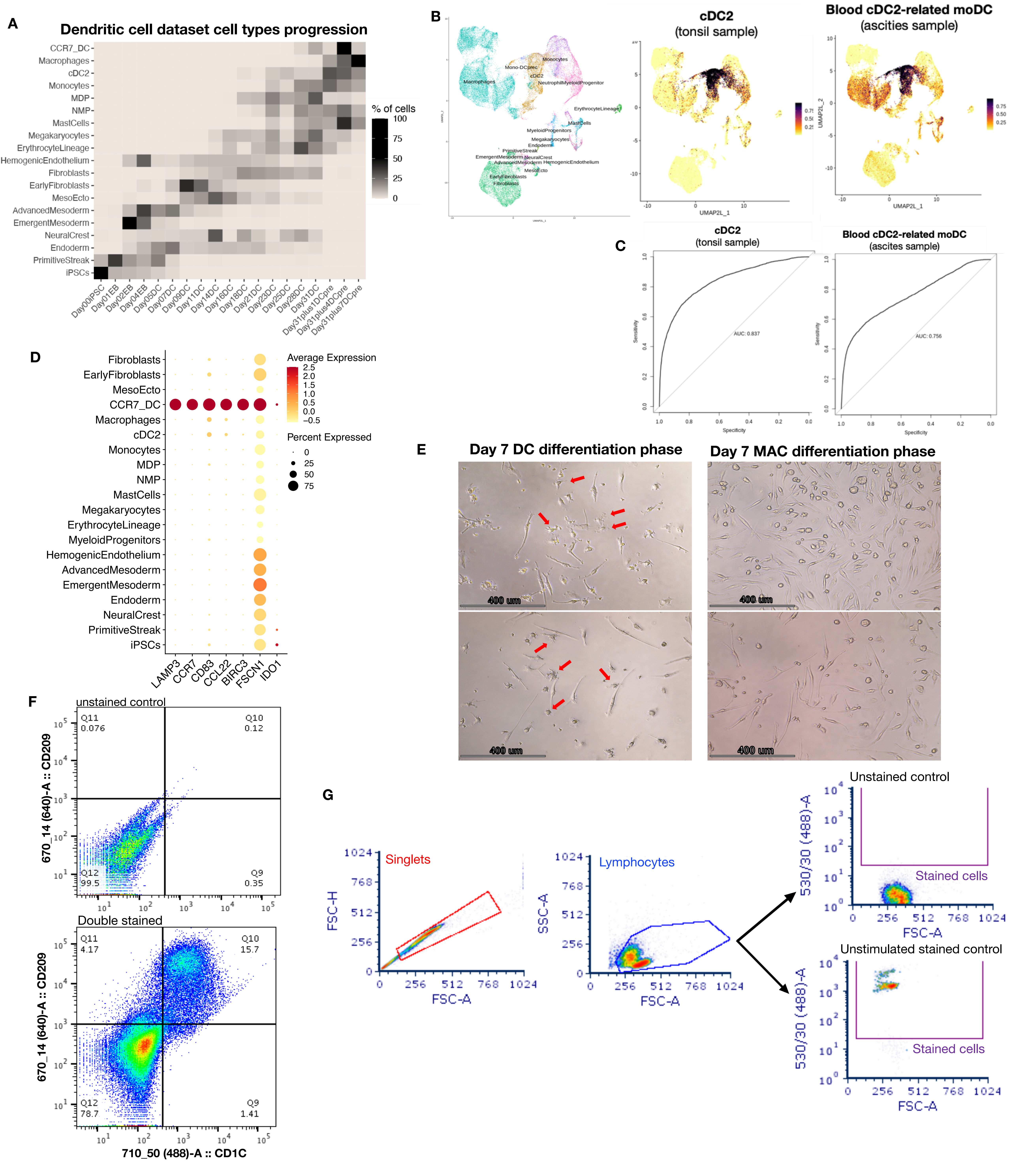

Supplementary Figure 9. Dendritic cells protocol

A, Heatmap of the percentage of cells distribution across all time points for each cell type in the DC dataset. **B,** Mixed macrophage and dendritic cell protocol UMAP corresponding to cells at time points from day31 until day31+7. Logistic regression prediction probabilities for models trained on adult tonsil cDC2 and two subsets of monocyte derived DCs[51]. **C,** Area under the curve (AUC) plots for the prediction probabilities of A in cells annotated as cDC2. **D,** Dot plot of CCR7+ DCs marker genes[52] expression in the DC dataset cell types. **E,** Representative images of the cells at the end of the dendritic cell and macrophage differentiation protocols. Red arrows highlight cells with dendrite-like structures. These were consistently observed at day7 of the DC differentiation phase n>3. **F,** FACS plots showing CD209 and CD1C marker protein expression levels for cells collected at the end of the differentiation of the DC protocol (day7 of the DC differentiation phase). **G,** Representative gating strategy for T cell activation FACS analysis.

ICAM1

>NC_000019.10:10271120-10286615 Homo sapiens chromosome 19, GRCh38.p13 Primary Assembly

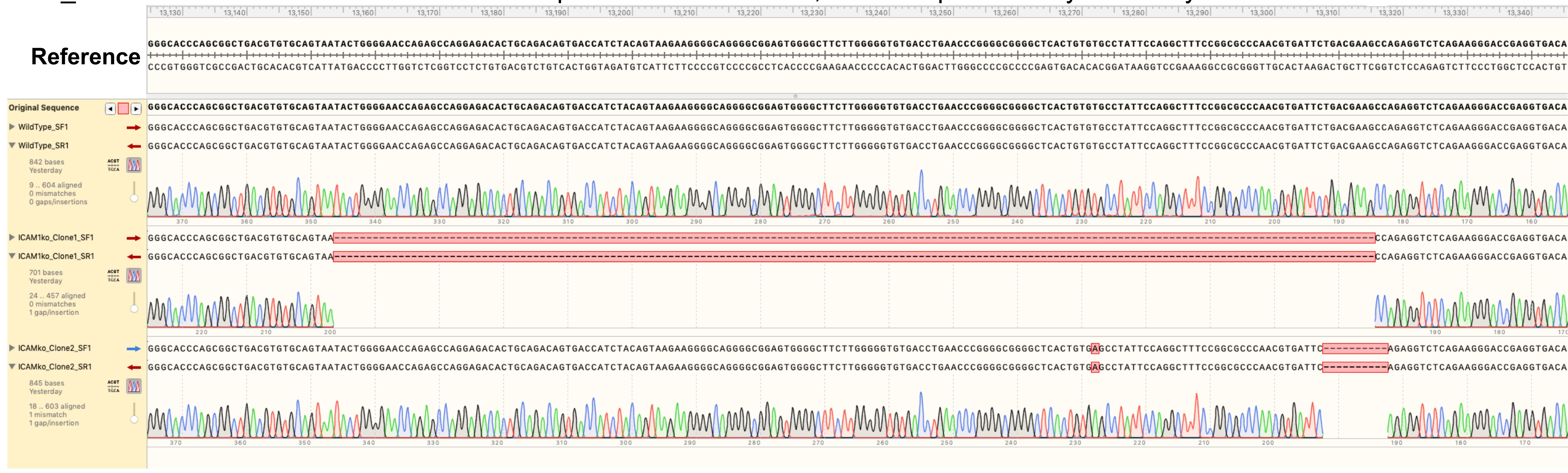

LSP1

>NC_000011.10:1853084-1892263 Homo sapiens chromosome 11, GRCh38.p13 Primary Assembly

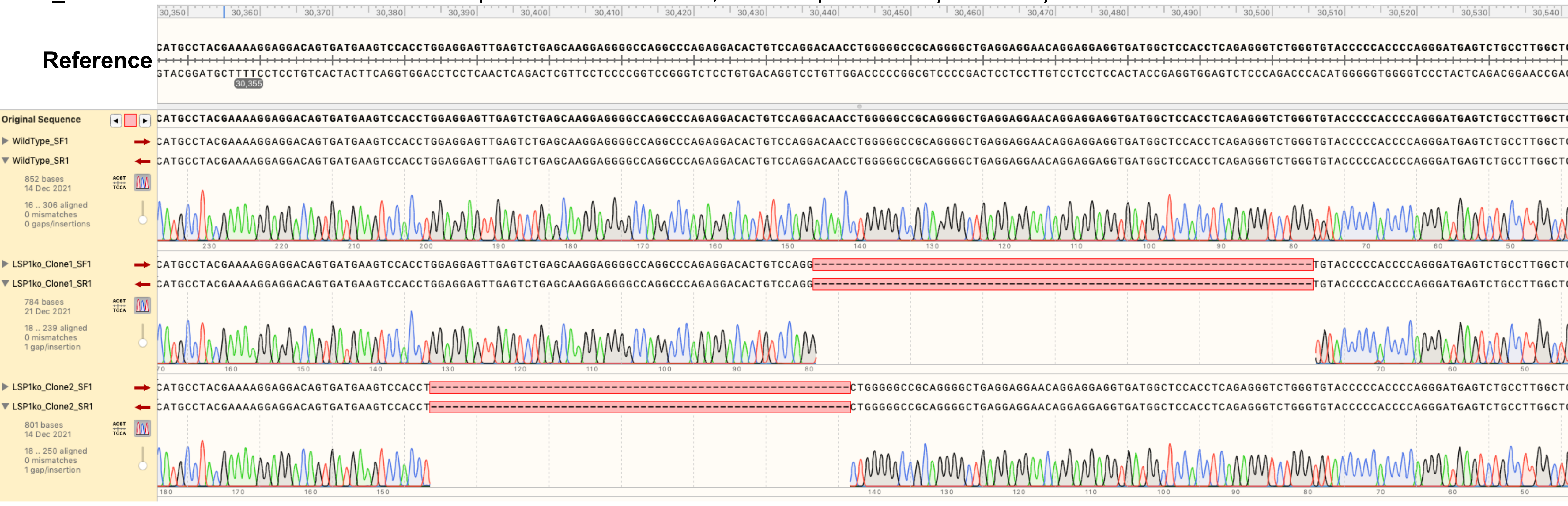

PRKCB

>NC 000016.10:23835983-24220611 Homo sapiens chromosome 16, GRCh38.p13 Primary Assembly

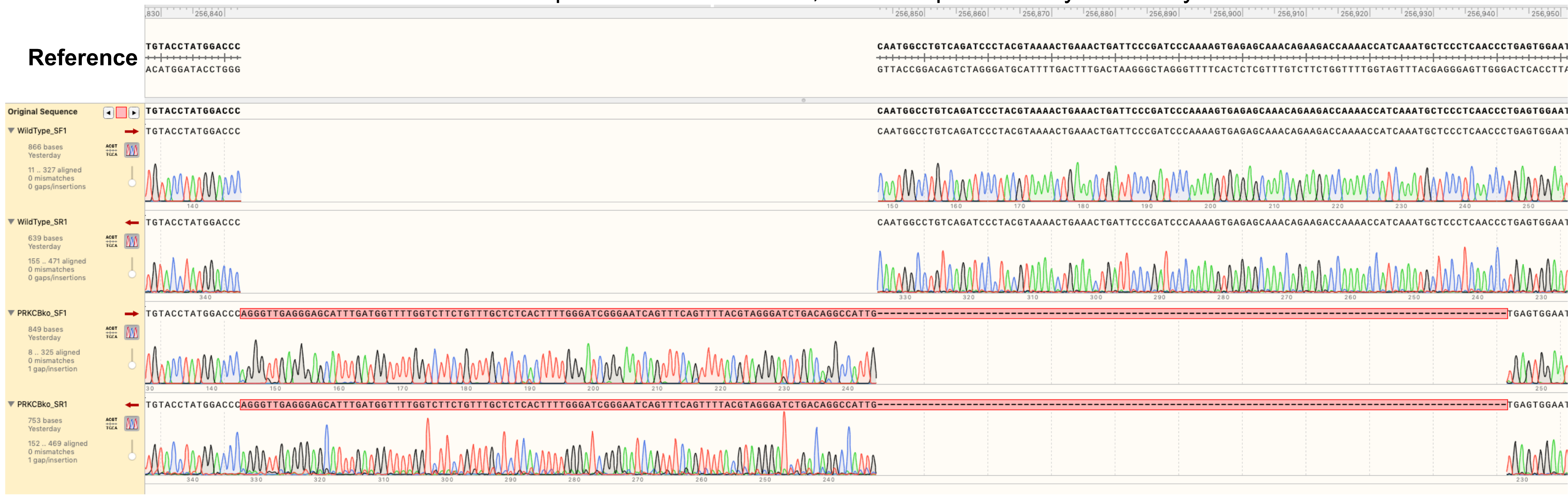

ZEB2

>NC_000002.12:c144520119-144384081 Homo sapiens chromosome 2, GRCh38.p13 Primary Assembly

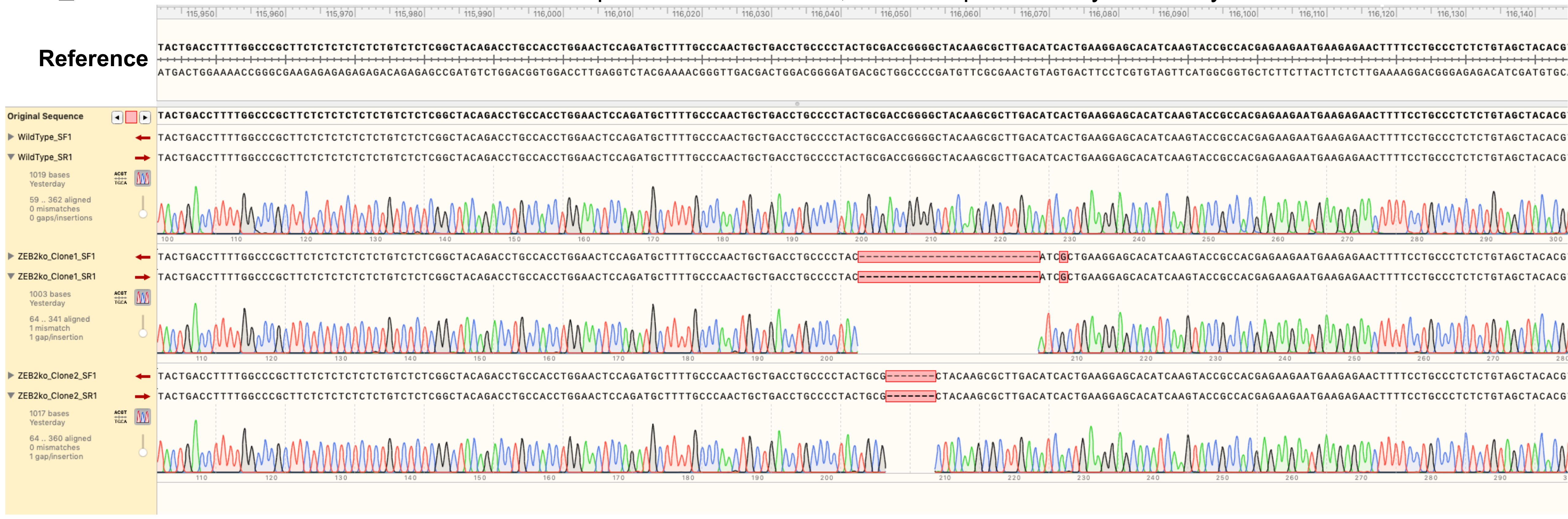

Supplementary Figure 10. Sanger Sequencing results from KO lines

For each KO gene, from top to bottom, there is the NCBI fasta file header for the genomic sequence used as a reference. Below is the reference genomic region matching the CRISPR targeted site, coordinates in grey refer to fasta file sequence stated above. Following below, there are the aligned sanger sequencing results, using both forward (SF1) and reverse (SR1) primers, for the wild type control cell line and the KO clones. Sanger sequencing results are shown as the sequences obtained for both primers in addition to the chromatogram for one of them. Differences from the reference are highlighted in red on the sanger results sequences. Only the sequence region around the expected targeting site are shown, the left yellow column contains a summary of the total number of mismatches and gaps/insertions found in the full sanger sequencing results.

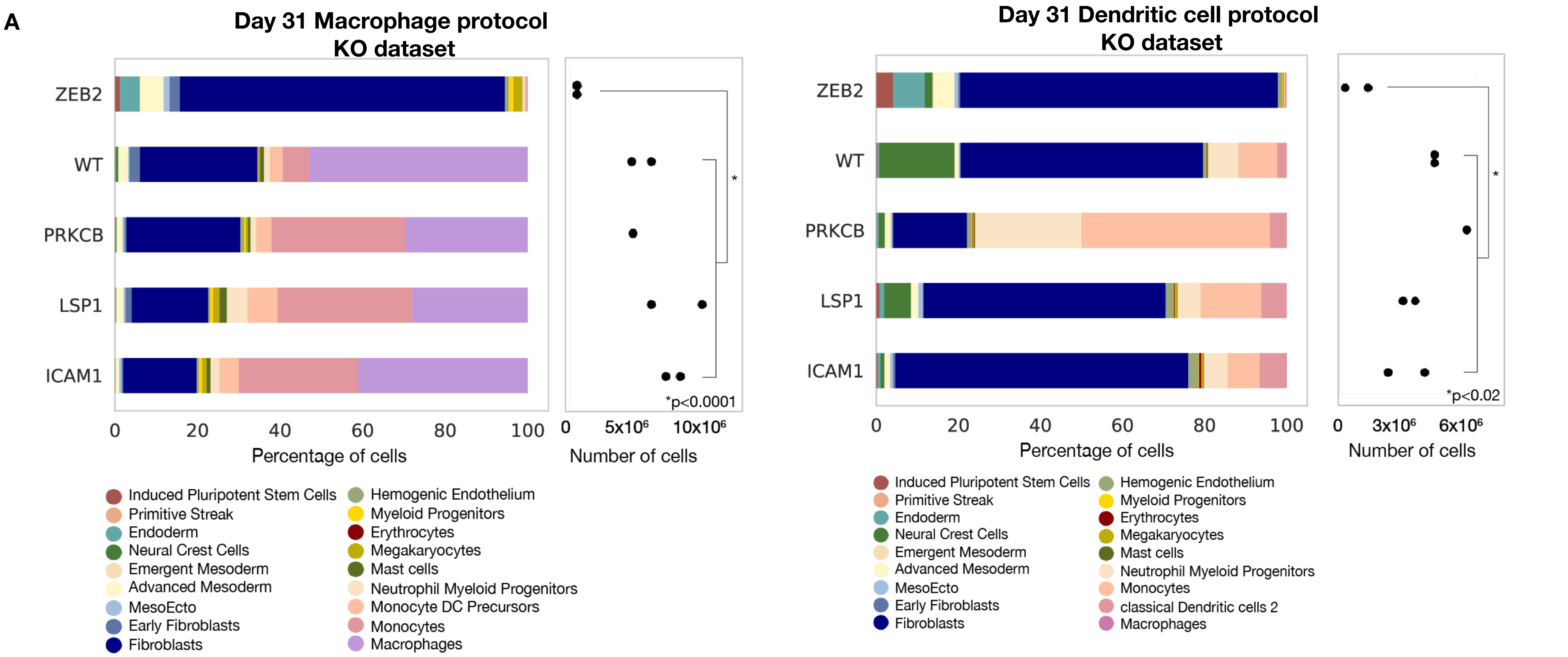

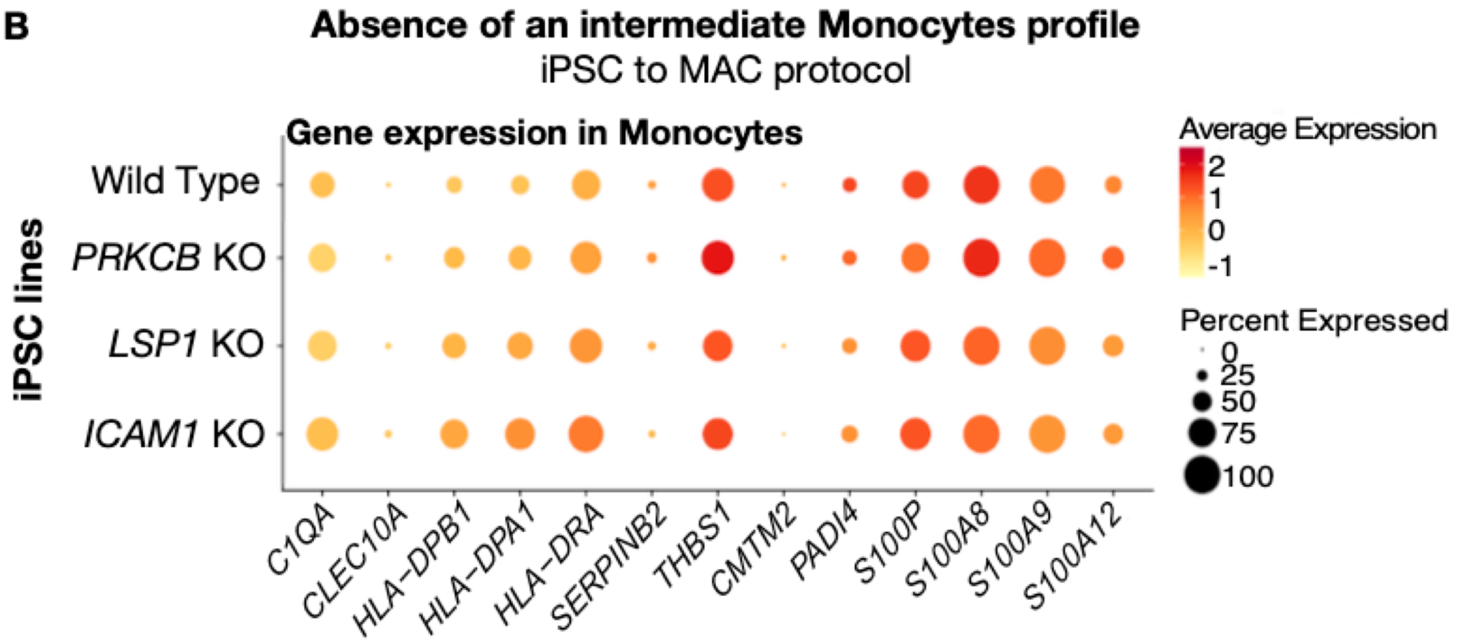

Supplementary Figure 11. Knock-out cell types and transcriptomic profiles

A, Stacked bar plots of the proportions of each cell type for each KO gene and WT lines on day31 cells for the macrophage (left) and dendritic cells (right) protocols. Adjacent plots show the number of cells collected for each cell line at day31, p-values for a two-sided Wilcox-text are shown. **B,** Dot plot with scaled gene expression levels in monocytes from WT and knock-out lines differentiated with the macrophage protocol. Genes shown are characteristic of intermediate monocytes and were significantly dysregulated in LSP1 and ICAM1 monocytes produced in the DC protocol.

Reagent/Materials table. Detailed information about all products mentioned on the Methods section

Reagent/Material in alphabetical order (full name)	CatalogNum	Supplier	Comment
96 Well Round Bottom Ultra Low Attachment Microplate	7007	CORNING	differentiation cell culture
BD Cytofix/Cytoperm kit	554714	ThermoFisher	FACS analysis
BMP4 (Recombinant Human BMP4)	120-05ET	PeproTech	differentiation cell culture
Brefeldin A	B7651	Sigma Aldrich	LPS experiment
BSA (Bovine Serum Albumin)	SH30574.02	GE Healthcare	single cell analysis
CFSE (eBioscience™ CFSE)	65-0850-84	ThermoFisher	T cell activation assay
Chromium Single Cell 3’ Kit v2	PN-120237	10X Genomics	single cell analysis
Chromium Single Cell 3’ Kit v3	PN-1000075	10X Genomics	single cell analysis
Chromium Single Cell ATAC Kit	PN-1000110	10X Genomics	single cell analysis
DPBS no calcium no magnesium	14190094	ThermoFisher	single cell analysis
DQ-OVA (DQ ovalbumin)	D-12053	ThermoFisher	FACS analysis
E8 (TeSR™-E8™ Kit)	5990	StemCell Technologies	differentiation cell culture
EasySep® human naïve CD4+ T cell isolation kits II	17555	StemCell Technologies	T cell activation assay
EDTA (UltraPure 0.5M EDTA solution)	15575-020	ThermoFisher	single cell analysis
FBS (Fetal Bovine Serum, qualified, heat inactivated)	10500064	ThermoFisher	differentiation cell culture
Ficoll-Paque PLUS	17144002	GE Healthcare	T cell activation assay
FLT3L (Recombinant Human Flt3-L)	300-19	PeproTech	differentiation cell culture
Gelatin solution, tissue culture grade	G1393-100ML	Sigma Aldrich	differentiation cell culture
GMCSF (Recombinant Human GM-CSF)	300-03	PeproTech	differentiation cell culture
Human Stem Cell Nucleofector® Kit 2	VPH-5022	Lonza	CRISPR knock outs
IL3 (Recombinant Human IL-3)	200-03	PeproTech	differentiation cell culture
IL34 (Recombinant Human IL-34)	200-34	PeproTech	differentiation cell culture
IL4 (Recombinant Human IL-4)	200-04	PeproTech	differentiation cell culture
Lidocaine hydrochloride monohydrate	L5647	Sigma Aldrich	single cell analysis
LPS (E.coli LPS O26:B6)	L8274	Sigma Aldrich	LPS experiment
MCSF (Recombinant Human M-CSF)	300-25	PeproTech	differentiation cell culture
Microplate 6 well clear flat bottom TC-treated	3506	Corning	differentiation cell culture
Puromycin	ant-pr-1	Invivogen	CRISPR knock outs
ROCK inh (Y-27362 dihydrochloride)	Y0503	Sigma Aldrich	differentiation cell culture
RPMI 1640 Medium, GlutaMAX™	61870036	ThermoFisher	differentiation cell culture
SCF (Recombinant Human SCF)	300-07	PeproTech	differentiation cell culture
StemPro -34 SFM	10639011	Life Technologies	differentiation cell culture
TrypLE Express Enzyme	12604013	Life Technologies	single cell analysis
V-PLEX Proinflammatory Panel 1 Human Kit	K15049D	Mesoscale	LPS experiment
VEGF (Recombinant Human VEGF121)	100-20A	PeproTech	differentiation cell culture
Vitronectin (VTN-N) Recombinant Human Protein, Truncated	A14700	Life Technologies	differentiation cell culture

Antibodies table. Detailed information about all antibodies used including clone, lot and dilution

Antibody	Brand	CatalogNum	Clone	Lot	Concentration
FCER1A	BD Biosciences	566608	AER-37	1037384	1/50 in 100ul for 1 million cells
CD117	eBioscience	17-1178-41	104D2	2333702	1/25 in 100ul for 1 million cells
CD1C	eBioscience	46-0015-42	L161	4336341	1/50 in 100ul for 1 million cells
CD209	Biolegend	330107	9E9A8	B228986	1/50 in 100ul for 1 million cells
CD11c	Biolegend	301608	3.9	B234325	1/50 in 100ul for 1 million cells
HLA-DR	Biolegend	307606	L243	B242529	1/50 in 100ul for 1 million cells
CD86	Biolegend	305414	IT2.2	B243405	1/50 in 100ul for 1 million cells
CD14	Biolegend	301808	M5E2	B222744	1/100 in 100ul for 1 million cells
CD64	Biolegend	305022	10.1	B213860	1/100 in 100ul for 1 million cells
CD3	Tonbo Biosciences	70-0037-U100	OKT3	P0037082420703	100 ng for 1 million of cells
Anti-CD3/CD28 (ImmunoCult)	StemCell Technologies	10971	NA	1000058646	6ul for 1 million of cells

Primers table. Primer sequences for sanger sequencing of human iPSC knock out loci

Primer	5' to 3' sequence
ICAM1_PF1	AGACCTTTGGTGAGGATTGAAGAAG
ICAM1_PR1	GAAAGTGCCATCCTTTAGACACTTG
ICAM1_SF1	GGGGAATGAAATGCCCCAGA
ICAM1_SR1	TCACACAGGACACGAAGCTC
ZEB2_PF1	TTGAGACAAGTAAGCAAAAAGAGGG
ZEB2_PR1	TCACACTGTGAGTTCCTGAGTAATC
ZEB2_SF1	CACACTCATTCGATCGTGAACA
ZEB2_SR1	ACAAGTCTGTCCTGGGCATG
PRKCB_PF1	TGGGCTTTGTGGAAAATAAGCTTAG
PRKCB_PR1	TTGGGAATAAAATGAGCCGATGAAG
PRKCB_SF1	TGCACTGCTTCAGCTATGGA
PRKCB_SR1	CCACACCACAGGCATTCTCA
LSP1_PF1	TTCGAGAATTTCCCATCTTCTCCTC
LSP1_PR1	AACAAATGCTGTTCCTTCCTGAG
LSP1_SF1	GTCTCAGATCCCTTGGCACT
LSP1_SR1	CTGCAAGGTCTAGGGGTGTG

P: PCR primer (amplification before sequencing)
S: Sanger sequencing primer
F: Forward primer
R: Reverse primer
