# Supplementary material for: Robust temporal map of human in vitro myelopoiesis using single-cell genomics
Source: Nat Commun. 2022 May 24;13:2885. doi: 10.1038/s41467-022-30557-4 (PMC9130280; doi:10.1038/s41467-022-30557-4)
Supplement: Supplementary file 2 — Description of Additional Supplementary Files [file 41467_2022_30557_MOESM2_ESM.pdf]

Description of Additional Supplementary Files

File Name: Supplementary Data 1
Description: Summary of 10X Genomics Chromium samples

File Name: Supplementary Data 2
Description: Summary of in vivo public datasets used

File Name: Supplementary Data 3
Description: Mean prediction probabilities for each LR model (rows) in each in vitro cell type cluster (columns) for the Discovery dataset

File Name: Supplementary Data 4
Description: Mean label transfer score for each in vitro validation dataset cell type cluster

File Name: Supplementary Data 5
Description: Transcription factor activities along trajectories in the macrophage protocol in vivo vs in vitro

File Name: Supplementary Data 6
Description: Transcription factor activities across samples in macrophage differentiation phase. Time points experiment.

File Name: Supplementary Data 7
Description: Differentially expressed genes between LPS and their respective control

File Name: Supplementary Data 8
Description: Mean Prediction score for each LR model (rows) in each in vitro cell type cluster (columns) for the DC dataset

File Name: Supplementary Data 9
Description: Transcription factor activities along trajectories in the DC protocol in vivo vs in vitro

File Name: Supplementary Data 10
Description: Proportion of cells for each KO and WT lines

File Name: Supplementary Data 11
Description: Differentially expressed genes between KO and WT genes
