# Supplementary material for: Robust temporal map of human in vitro myelopoiesis using single-cell genomics
Source: Nat Commun. 2022 May 24;13:2885. doi: 10.1038/s41467-022-30557-4 (PMC9130280; doi:10.1038/s41467-022-30557-4)
Supplement: Supplementary file 4 — Reporting Summary [file 41467_2022_30557_MOESM4_ESM.pdf]

nature portfolio

Corresponding author(s): Clara Alsinet
Daniel J Gaffney
Damiana Alvarez-Errico
Roser Vento-Tormo

Last updated by author(s): Apr 22, 2022

Reporting Summary

Nature Portfolio wishes to improve the reproducibility of the work that we publish. This form provides structure for consistency and transparency in reporting. For further information on Nature Portfolio policies, see our Editorial Policies and the Editorial Policy Checklist.

Statistics

For all statistical analyses, confirm that the following items are present in the figure legend, table legend, main text, or Methods section.

n/a	Confirmed	
☐	☒	The exact sample size (*n*) for each experimental group/condition, given as a discrete number and unit of measurement
☐	☒	A statement on whether measurements were taken from distinct samples or whether the same sample was measured repeatedly
☐	☒	The statistical test(s) used AND whether they are one- or two-sided<br>*Only common tests should be described solely by name; describe more complex techniques in the Methods section.*
☒	☐	A description of all covariates tested
☐	☒	A description of any assumptions or corrections, such as tests of normality and adjustment for multiple comparisons
☐	☒	A full description of the statistical parameters including central tendency (e.g. means) or other basic estimates (e.g. regression coefficient) AND variation (e.g. standard deviation) or associated estimates of uncertainty (e.g. confidence intervals)
☐	☒	For null hypothesis testing, the test statistic (e.g. *F*, *t*, *r*) with confidence intervals, effect sizes, degrees of freedom and *P* value noted<br>*Give P values as exact values whenever suitable.*
☒	☐	For Bayesian analysis, information on the choice of priors and Markov chain Monte Carlo settings
☒	☐	For hierarchical and complex designs, identification of the appropriate level for tests and full reporting of outcomes
☐	☒	Estimates of effect sizes (e.g. Cohen's *d*, Pearson's *r*), indicating how they were calculated

Our web collection on statistics for biologists contains articles on many of the points above.

Software and code

Policy information about availability of computer code

Data collection	No specific software was used for data collection.
Data analysis	All data analysis was performed using Cell Ranger v3.1.0, R v4.0 and Python v3.8. Main packages include R packages Seurat v3.2.2 and v 4.0.2, Signac v1.1.1, DoRothEA v1.2.1 and python package scVelo v0.2.2. All relevant code is available in https://github.com/Ventolab/iPSCmyeloid. For FACS data analysis we used FlowJo v10.

For manuscripts utilizing custom algorithms or software that are central to the research but not yet described in published literature, software must be made available to editors and reviewers. We strongly encourage code deposition in a community repository (e.g. GitHub). See the Nature Portfolio guidelines for submitting code & software for further information.

Data

Policy information about availability of data

All manuscripts must include a data availability statement. This statement should provide the following information, where applicable:

- Accession codes, unique identifiers, or web links for publicly available datasets
- A description of any restrictions on data availability
- For clinical datasets or third party data, please ensure that the statement adheres to our policy

Raw data has been uploaded inArrayExpress (E-MTAB-11623 for single cell RNAseq and E-MTAB-11616 for ATACseq), and can be accessed and downloaded through the web portal www.HiPImmuneatlas.org. Single cell sequencing data was mapped against GRCh38 http://ftp.ensembl.org/pub/release-100/. Publicly available datasets used include Gastrulation http://www.human-gastrula.net/, Fetal liver (+kidney +skin) E-MTAB-7407, Fetal thymus E-MTAB-8581, Placenta E-MTAB-6701, Oncofetal HCC https://doi.org/10.17632/6wmzcskt6k.1, Yolk sac GSE133345 and adult DCs and Macs GSE115006.

Field-specific reporting

Please select the one below that is the best fit for your research. If you are not sure, read the appropriate sections before making your selection.

☒ Life sciences ☐ Behavioural & social sciences ☐ Ecological, evolutionary & environmental sciences

For a reference copy of the document with all sections, see nature.com/documents/nr-reporting-summary-flat.pdf

Life sciences study design

All studies must disclose on these points even when the disclosure is negative.

Sample size	Sample size was not calculated following any specific statistical test. Three to 6 cell lines were used for most experiments, these numbers are sufficient to discard the effect of outliers. We used only one line in KO and stimulation experiments, in all cases isogenic/negative controls are used to draw conclusions.
Data exclusions	All datasets are provided in full in public databases and only low quality single cell RNAseq samples or single cell RNAseq cells were excluded from final analysis following pre-defined standard thresholds. These are clearly described.
Replication	All results shown were replicated in 2 or more experiments and number of replicates is stated for each experiment.
Randomization	No randomization was used in this study since all lines came from healthy donors and no covariates have been described to impact myeloid iPSC differentiation.
Blinding	Investigators were not blinded, standard good laboratory practices and analysis employed avoid the need for blinding.

Reporting for specific materials, systems and methods

We require information from authors about some types of materials, experimental systems and methods used in many studies. Here, indicate whether each material, system or method listed is relevant to your study. If you are not sure if a list item applies to your research, read the appropriate section before selecting a response.

Materials & experimental systems

n/a	Involved in the study	
☐	☒	Antibodies
☐	☒	Eukaryotic cell lines
☒	☐	Palaeontology and archaeology
☒	☐	Animals and other organisms
☒	☐	Human research participants
☒	☐	Clinical data
☒	☐	Dual use research of concern

Methods

n/a	Involved in the study	
☒	☐	ChIP-seq
☐	☒	Flow cytometry
☒	☐	MRI-based neuroimaging

Antibodies

Antibodies used

Antibody Brand Clone CatalogNum Lot Concentration
FCER1A BD Biosciences AER-37 566608 1037384 1/50 in 100ul for 1 million cells
CD117 eBioscience 104D2 17-1178-41 2333702 1/25 in 100ul for 1 million cells
CD1C eBioscience L161 46-0015-42 4336341 1/50 in 100ul for 1 million cells
CD209 Biolegend 9E9A8 330107 B228986 1/50 in 100ul for 1 million cells
CD11c Biolegend 3.9 301608 B234325 1/50 in 100ul for 1 million cells
HLA-DR Biolegend L243 307606 B242529 1/50 in 100ul for 1 million cells
CD86 Biolegend IT2.2 305414 B243405 1/50 in 100ul for 1 million cells
CD14 Biolegend M5E2 301808 B222744 1/100 in 100ul for 1 million cells
CD64 Biolegend 10.1 305022 B213860 1/100 in 100ul for 1 million cells

Validation

FCER1A
Two-parameter flow cytometric analysis of FcεR1α expression on human peripheral blood leucocyte populations. Human whole blood was stained with either PE Mouse IgG2b, κ Isotype Control (Cat. No. 555058; Left Plot) or PE Mouse Anti-Human FcεR1α antibody (Cat. No. 566607/566608; Right Plot) at 1 µg/test. Erythrocytes were lysed with BD Pharm Lyse™ Lysing Buffer (Cat. No. 555899). Two-parameter flow cytometric pseudo color plots showing the correlated expression of FcεR1α (or Ig Isotype control staining) versus side light-scatter signals (SSC-A) were derived from gated events with the forward and side light-scatter characteristics of viable leucocyte populations. Flow

cytometric analysis was performed using a BD FACSCelesta™ Flow Cytometer System. Data shown on this Technical Data Sheet are not lot specific.

CD117
This 104D2 antibody has been pre-titrated and tested by flow cytometric analysis of normal human peripheral blood cells. This can be used at 5 µL (0.25 µg) per test. A test is defined as the amount (µg) of antibody that will stain a cell sample in a final volume of 100 µL. Cell number should be determined empirically but can range from 10^5 to 10^8 cells/test.

CD1C
This L161 antibody has been pre-titrated and tested by flow cytometric analysis of normal human peripheral blood cells. This can be used at 5 µL (0.06 µg) per test. A test is defined as the amount (µg) of antibody that will stain a cell sample in a final volume of 100 µL. Cell number should be determined empirically but can range from 10^5 to 10^8 cells/test.

CD209
APC Mouse IgG2a, κ Isotype Ctrl for flow cytometry. Each lot of this antibody is quality control tested by immunofluorescent staining with flow cytometric analysis https://www.biolegend.com/protocols/cell-surface-flow-cytometry-staining-protocol/4283/. For flow cytometric staining, the suggested use of this reagent is 5 µl per million cells in 100 µl staining volume or 5 µl per 100 µl of whole blood.

CD11c
PE/Cyanine7 Mouse IgG1, κ Isotype Ctrl for flow cytometry. Each lot of this antibody is quality control tested by immunofluorescent staining with flow cytometric analysis https://www.biolegend.com/protocols/cell-surface-flow-cytometry-staining-protocol/4283/. For flow cytometric staining, the suggested use of this reagent is 5 µl per million cells in 100 µl staining volume or 5 µl per 100 µl of whole blood.

HLA-DR
PE Mouse IgG2a, κ Isotype Ctrl for flow cytometry. Each lot of this antibody is quality control tested by immunofluorescent staining with flow cytometric analysis https://www.biolegend.com/protocols/cell-surface-flow-cytometry-staining-protocol/4283/. For flow cytometric staining, the suggested use of this reagent is 5 µl per million cells in 100 µl staining volume or 5 µl per 100 µl of whole blood.

CD86
Alexa Fluor® 488 Mouse IgG2b, κ Isotype Ctrl for flow cytometry. Each lot of this antibody is quality control tested by immunofluorescent staining with flow cytometric analysis https://www.biolegend.com/protocols/cell-surface-flow-cytometry-staining-protocol/4283/. For flow cytometric staining, the suggested use of this reagent is 5 µl per million cells in 100 µl staining volume or 5 µl per 100 µl of whole blood.

CD14
APC Mouse IgG2a, κ Isotype Ctrl for flow cytometry. Each lot of this antibody is quality control tested by immunofluorescent staining with flow cytometric analysis https://www.biolegend.com/protocols/cell-surface-flow-cytometry-staining-protocol/4283/. For flow cytometric staining, the suggested use of this reagent is 5 µl per million cells in 100 µl staining volume or 5 µl per 100 µl of whole blood.
CD64
PPE/Cyanine7 Mouse IgG1, κ Isotype Ctrl for flow cytometry. Each lot of this antibody is quality control tested by immunofluorescent staining with flow cytometric analysis https://www.biolegend.com/protocols/cell-surface-flow-cytometry-staining-protocol/4283/. For flow cytometric staining, the suggested use of this reagent is 5 µl per million cells in 100 µl staining volume or 5 µl per 100 µl of whole blood.

Eukaryotic cell lines

Policy information about cell lines

Cell line source(s)	All cell lines used came from the HiPSci.
Authentication	All cell lines have been genome sequenced.
Mycoplasma contamination	All batches of frozen cells were mycoplasma tested.
Commonly misidentified lines (See ICLAC register)	*Name any commonly misidentified cell lines used in the study and provide a rationale for their use.*

Flow Cytometry

Plots

Confirm that:

☒ The axis labels state the marker and fluorochrome used (e.g. CD4-FITC).

☒ The axis scales are clearly visible. Include numbers along axes only for bottom left plot of group (a 'group' is an analysis of identical markers).

☒ All plots are contour plots with outliers or pseudocolor plots.

☒ A numerical value for number of cells or percentage (with statistics) is provided.

Methodology

Sample preparation	Samples were detached from tissue cultured plates, washed with DPBS and stained in DPBS+FBS FACS solution
Instrument	Becton Dickinson LSRFortessa II
Software	FlowJo and FCS Express
Cell population abundance	Samples were not sorted, just analysed. Full/live cells and singlets were selected for analysis and these represented between 90 and 99%+ of cells for all analysis shown in the manuscript.
Gating strategy	FSC/SSC was used for gating on full/live cells vs debris/dead cells and these were further selected for singlets using FSC-W/FSC-A. For the T cell activation experiment FSC-H/FSC-A was used to select singlets and FSC-A/SSC-A to select lymphocytes, later 530/30 (488)-A / FSC-A was used to exclude unstained myeloid cells. Results plots shown are contour plots or histograms depending on type of analysis reported.

☒ Tick this box to confirm that a figure exemplifying the gating strategy is provided in the Supplementary Information.
